# Nucleotide resolution 4-thiouridine sequencing by SNU-Seq and sf4sU-Seq reveals the transcriptional responsiveness of an epigenetically primed human genome

**DOI:** 10.1093/nar/gkag652

**Published:** 2026-06-27

**Authors:** Umut Gerlevik, Philipp Lorenz, Anna Lamstaes, Harry Fischl, Shidong Xi, Aksel Saukko-Paavola, Struan Murray, Thomas Brown, Alexander Welch, Charlotte George, Andrew Angel, Andre Furger, Jane Mellor

**Affiliations:** Department of Biochemistry, University of Oxford, South Parks Road, Oxford OX1 3QU, UK; Faculty of Medicine, Ludwig-Maximilians-Universität München, Munich D-80539, Germany; Department of Biochemistry, University of Oxford, South Parks Road, Oxford OX1 3QU, UK; Department of Biochemistry, University of Oxford, South Parks Road, Oxford OX1 3QU, UK; Department of Biochemistry, University of Oxford, South Parks Road, Oxford OX1 3QU, UK; Department of Biochemistry, University of Oxford, South Parks Road, Oxford OX1 3QU, UK; Department of Biochemistry, University of Oxford, South Parks Road, Oxford OX1 3QU, UK; Department of Biochemistry, University of Oxford, South Parks Road, Oxford OX1 3QU, UK; Department of Biochemistry, University of Oxford, South Parks Road, Oxford OX1 3QU, UK; Department of Biochemistry, University of Oxford, South Parks Road, Oxford OX1 3QU, UK; Department of Biochemistry, University of Oxford, South Parks Road, Oxford OX1 3QU, UK; Department of Biochemistry, University of Oxford, South Parks Road, Oxford OX1 3QU, UK; School of Natural and Computing Sciences, University of Aberdeen, Aberdeen AB24 3UE, UK; Department of Biochemistry, University of Oxford, South Parks Road, Oxford OX1 3QU, UK; Department of Biochemistry, University of Oxford, South Parks Road, Oxford OX1 3QU, UK

## Abstract

Genomes are pervasively transcribed, leading to stable and unstable transcripts that influence 3-dimensional genome organization and gene regulation. High sensitivity and nucleotide resolution are required to resolve mammalian transcriptomes. Here, we exploit the sensitivity of 4-thiouridine (4sU) in two nucleotide-resolution methods: Single-Nucleotide resolution 4sU sequencing (SNU-Seq) and size-fractionated 4sU-Seq (sf4sU-Seq). sf4sU-Seq involves gel isolation of abundant 4sU-labelled promoter-proximal transcripts, enabling nucleotide resolution mapping of transcription start sites and promoter-proximal pauses (PPPs) around +63 nucleotides on Pol II-transcribed loci. SNU-Seq maps the precise position of polymerases on transcription units, including paused Pol II at the PPP, validated using sf4sU-Seq, and enables the discovery of thousands of divergently transcribed intragenic and intergenic regions of open chromatin, many uncharacterized. Conversely and consistent with extensive epigenetic priming, hundreds of the >10 000 regions of acetylated open chromatin lacking detectable transcription using SNU-Seq, show IFNγ-dependent induction of divergent transcription, linked at selected loci to the formation of promoter-enhancer loops. At other primed regions, formation of promoter–enhancer loops is coincident with divergent transcription at the enhancer but precedes transcription of pre-mRNA from the promoter, supporting distinct priming mechanisms. Thus, 4sU-based methods, coupled to chromatin analysis, enable detailed characterization of genome structure, transcription, and responsiveness.

## Graphical Abstract

**Graphical Abstract:**
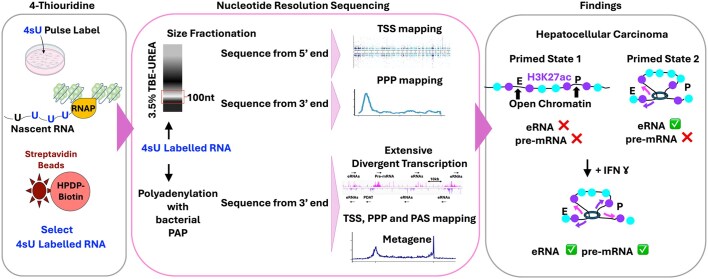

## Introduction

Genomes are pervasively transcribed, leading to stable and unstable transcripts that influence 3-dimensional genome organization [1, 2], the epigenome and gene regulation [3]. Transcription of unstable transcripts impacts local and higher-order chromatin structures [2, 4], simply through the act of transcription, through retained nascent transcripts potentiating the formation of DNA:RNA hybrid R-loops or by recruiting functional RNA-binding proteins potentiating new combinatorial responses to environmental signals and, if mis-regulated in disease, often through transcription-DNA replication conflict [5]. In addition, some unstable transcripts have functional impacts in the cytoplasm by influencing messenger RNA (mRNA) stability, mRNA translation, or the composition of the translatome [6]. Routine mapping of precisely where and when transcription occurs gives insights into co-transcriptional events during the production and processing of non-coding transcripts and pre-mRNAs, including the promoter-proximal pause (PPP) [7] and alternative polyadenylation site (PAS) usage [8, 9], and how these relate to epigenomic features.

As the sensitivity and resolution of methods for mapping where and when genomes are transcribed have improved, so has our understanding of the abundance of, and range of functions associated with, unstable transcripts [10]. Mapping and functional analysis has been aided by depletion of individual components of the machineries associated with transcription cycles [11–13], including mediator, a coordinator of initiation, elongation, and the epigenome [14]; the kinase pTEF-b regulating release from the PPP coordinated with transcription initiation [15]; integrator [16] and restrictor [17] acting together to control sequence-independent promoter-proximal transcription termination particularly of non-coding transcripts; sequence dependent transcription termination by the Xrn2 complex linked the activity of the cleavage and polyadenylation complex [18, 19]; and the NEXT and PAXT complexes targeting exosome-dependent RNA decay [20].

These methods have led to the discovery of the non-coding exosome-targeted transcripts such as the divergently transcribed enhancer RNAs (eRNAs) [21–23], divergent transcripts upstream of pre-mRNAs known as PROMPTs (promoter upstream transcripts) or PDATs (promoter divergent antisense transcripts), whose levels vary with cellular stress and by transcription into promoter regions have the potential to control expression of nearby genes, and long non-coding transcripts [24, 25]. Although these approaches allow functional analysis, even fast depletion of factors may lead to stress responses in mammalian cells [26]; thus, it is preferable to characterize responses to environmental signals under native conditions.

There are four general approaches to map newly synthesized transcripts (reviewed in [10]), including enrichment of transcripts associated with RNA polymerase II (RNAPII or Pol II) (e.g. mNET-Seq) [25, 27–30], enrichment of chromatin-associated transcripts from the nucleus (e.g. ChrRNA-Seq) [31, 32], “run-on” approaches in permeabilized cells (e.g. PRO-Seq) [7, 33–36], and metabolic pulse-labelling coupled with affinity purification of transcripts produced or processed during the 4sU pulse (e.g. 4sU-Seq, TT-Seq) [37–43]. Each method produces distinct metagene profiles across genes, consistent with each technique capturing different aspects of transcription, although some are challenging to conduct and/or lack base pair resolution [44, 45]. 4sU-Seq [43] and TT-Seq [39] have high sensitivity, as a short pulse of 4-thiouridine (4sU) [46] is rapidly taken up by cells, phosphorylated, and incorporated into transcripts, but lack base pair resolution as transcript processing, library preparation, and data smoothing reduce resolution to about 200 nt (TT-Seq) or introduce a 5′ bias (4sU-Seq), and the co-transcriptional events of interest here, such as PPP of Pol II and alternative PAS usage are not easily resolved by TT-Seq [39, 47]. The addition of an artificial poly(A) tail to the 3′ nucleotide of unfragmented 4sU-labelled transcripts synthesized/degraded during the 4sU pulse label offers a cost-effective and straightforward method for library preparation with standard oligo(dT) primer-based RNA-Seq library kits and commercially available 3′ RNA sequencing kits. Importantly, this approach generates nucleotide resolution data, as in Single-Nucleotide resolution 4sU-sequencing (SNU-Seq) in adherent mammalian cells (HEK293 and Hep3B) [48] or pA+/pA- 3′ end RNA-Seq applied to HeLa cells [13, 16, 49] and mouse embryonic stem cells (mESC) [50] based on a method originally developed in *Saccharomyces cerevisiae* [41]. However, the precise sources of reads obtained in mammalian cells are assumed to be similar to those determined in yeast [20] using similar methodology [41], despite marked differences in the mechanisms and control of PolII transcription.

Here, we applied a series of controls to determine the precise source of reads in mammalian cells, helping to resolve polyadenylation (PAS) and alternative polyadenylation sites (APA) [18], the extent of transcription after polyadenylation but before termination, and events in the promoter-proximal region included early termination linked to host polyadenylation [51] and negative elongation factor (NELF)-dependent pausing of Pol II with the 3′ nucleotide in its active site at the PPP [7, 52]. The development of size-fractionated 4sU-Seq (sf4sU-Seq), involving gel purification of short promoter-proximal 4sU-pulse labelled transcripts, enabled mapping of the transcription start site (TSS) and the putative PPP a median 63 nt downstream of the TSS on the same gene. To validate that the 3′ ends of these transcripts are coincident with the PPP, genes were stratified into six groups based on the shape of metagenes in the promoter-proximal regions. Genes with the most distinct PPP in mNET-Seq data, were also most enriched in sf4sU-Seq 3′ reads, the NELF, implicated in promoter-proximal pausing, and the Integrator complex that regulates the termination of non-productive Pol II transcription events, including around the promoter [16, 53–55], at eRNAs and small nuclear RNAs (snRNAs) [56]. Thus, mapping the precise source of reads using techniques such as sf4sU-Seq and SNU-Seq complements approaches such as factor immunoprecipitation [57] or knockdowns to explore dependent events around the promoter and at termination sites [13, 16, 55, 58, 59]. Finally, SNU-Seq was combined with chromatin analysis to monitor interferon gamma (IFNγ)-dependent temporal activation of divergent transcripts at enhancers and their long-range interactions with promoters in hepatocytes, revealing distinct epigenetic priming mechanisms.

## Materials and methods

A detailed version of materials, sources and methods is available in the Supplementary Data.

### Cell culture

HEK293 and Hep3B cells were cultured in Dulbecco's modified Eagle medium (DMEM), and supplemented with 10% (v/v) FBS and 1% (v/v) Penicillin–Streptomycin (Sigma cat #P0781). The cells were passaged 48 h before harvesting at 80% confluency. After passage, HEP3B cells were left for 24 h to adapt, then left untreated, or treated with 10 ng/ml of IFNγ for the time stated.

### TT-Seq

TT-Seq was performed as described in the original publication [39] with modifications detailed in the Supplementary Methods, including details of “Spike In” RNAs.

### Single-nucleotide 4sU-sequencing (SNU-Seq)

SNU-Seq was performed using the TT-Seq protocol but omitting the sonication step. Total RNA aliquots for 3′ RNA-Seq were prepared from the same samples used for SNU-Seq and first treated with DNase I and cleaned up using the Monarch Spin RNA Cleanup Kit (NEB T2040L). “No 4sU” libraries were prepared in the same way as SNU-Seq samples, except that water was added instead of 4sU. rRNA depletion was performed using the NEBNext rRNA Depletion kit for Human Cells (NEB E6310S) after streptavidin pulldown. 150 ng of thio-labelled RNAs were polyadenylated using the NEB *Escherichia coli* poly(A) polymerase (bPAP) for 45 min at 37°C. SNU-Seq libraries were prepared and sequenced by Lexogen GmbH (Vienna, Austria). Calibration of samples was achieved by calculating scaling factors from spike-in counts between samples via DESeq2, based on the RNA counts table generated by featureCounts. Bedgraph and bigwig files were generated from bam files using bedtools and wigToBigWig, respectively. Track files with 3′ end single-nucleotide coverage were generated by using the bedtools genomecov function’s “-3” option. A further normalization between +bPAP and no bPAP libraries was done by using 3′ UTR counts obtained using bedtools map function’s “-o sum” option. Details are provided in the Supplementary Methods.

### Size-fractionated 4sU-Seq

The thio-labelled RNA was generated as described for SNU-Seq and run on a 3.5% TBE-Urea gel (8 M urea). The small thio-labelled RNA gel region was cut using a razor blade and incubated at −80°C for 10 min, then extracted using dialysis. Agilent’s Bioanalyzer RNA Pico chip was used to assess the quality and size distribution of the size-selected RNA. The size-fractionated thio-labelled RNA was decapped with 5′ Pyrophosphohydrolase (NEB) in ThermoPol^®^ reaction buffer following the manufacturer’s instructions. Libraries were generated using the NEBNext Small RNA Library kit for Illumina following the kit instructions. Details of the data handling steps to generate 3′ end and 5′ end reads are provided in the Supplementary Methods.

### Annotation preparation for metagene analysis and mathematical modelling

For an unbiased metagene representation, the averaged regions are separated from other possible signals. GENCODE (v46) comprehensive gene annotations (*n* = 70 611) were filtered to retain genes within chr1 to chr22 to exclude the biased/unmapped regions of the reference genome (*n* = 59 950). Then, only genes with a minimum distance of 3.5 kb between them, regardless of the strand, were kept (*n* = 12 224). From this subset, only the protein-coding genes ≥1 kb were retained (*n* = 2807). Finally, UTRs within ±100 bp of a gene end with a transcript support level 1 to 5 were detected, and they were merged if multiple PAS/UTRs were present for a gene. Only the genes with a 3′ UTR annotation were kept (*n* = 2394). This is the GENCODE annotation subset that is used for metagene analysis and mathematical modelling.

### Mathematical modelling and determination of transcription constants

Synthesis rates (SR) and decay rates (DR) were determined based on a previously published modelling approach [39, 60]: DR = −(1/time)* log(1 − RNA_4sU_/RNA_total_), and SR = RNA_total_*DR, where time for labelling is 10 min, RNA_4sU_ is the normalized SNU-Seq gene body (TSS + 200 nt to the 3′ UTR start site) counts with bPAP treatment, exploiting the total RNA signal and the thio-labelled RNA signal from the same samples or HEK293 total or 3′ end RNA-Seq counts from [61]. The pausing index was calculated as described [57], using the ratio of normalized TSS (from −50 nt to + 200 nt) counts to gene body counts.

### Metagene analysis and visualization

deepTools was used for metagene calculation and visualization using the “scale-regions” mode to compute metagene matrices with a 5 kb gene body length and 2 kb flanking regions, a 20 nt bin size achieved by averaging. Missing or zero-valued data were skipped. Each strand was calculated separately with strand-specific annotations, then merged with “computeMatrixOperations rbind” before visualization. “computeMatrixOperations subset” was used to subset the matrix for the libraries of interest.

### Statistical data analysis

*P*-values were determined using the non-parametric Wilcoxon rank sum test. The Bonferroni correction for multiple testing was applied when required. For correlations, Pearson (*r*) was used for the correlation between repeats based on the counts, while Kendall (*τ*) was used for the correlation between synthesis rates and counts, and Spearman (*ρ*) was used in all other cases. Principal Component Analysis (PCA) and data visualization were performed using deepTools (multiBigwigSummary followed by plotPCA) or in R using the PCAtools and ggplot2 with ggpubr packages, respectively. To compare the splicing levels in SNU-Seq and total RNA libraries, SPLICE-q [62] was used to calculate splicing efficiency (SE) from the BAM files. The SE score refers to the number of splicing events based on the ratio of spliced to unspliced reads found around the splice junctions. The SE score (between 0 and 1) with values closer to 1 indicating a greater number of splicing events. The mean SE per library was computed to improve comparisons between libraries. To investigate the effect of the background signal (no 4sU) and the host polyadenylation signal during the pulse labelling window (no bPAP), a signal subtraction was performed for each nucleotide in the genome. For this exhaustive subtraction, deepTools bigwigCompare was utilized with “–operation subtract –binSize 1” parameters.

### Nuclear and cytoplasmic RNA extraction and PAS mapping

Extraction of RNA from nuclear and cytoplasmic subcellular fractions of HEK293 cells (*n* = 3) was as described [63]. Human poly(A) site (PAS) annotations were obtained from PolyA_DB3 [64]. Each PAS was extended 20 nt 3′ and 200 nt 5′ from the site of cleavage, and those that overlapped on the same strand after extension were combined into a single PAS annotation. Mapped reads were narrowed to their 3′ most nucleotide. Those overlapping the extended PAS annotations were counted omitting PASs associated with non-coding RNAs and genes not in the RefSeq gene database [65]. Genes with only one PAS were also excluded.

### Chromatin analysis in Hep3B cells

ATAC-Seq (tagmentation) was performed on 5 × 10^6^ Hep3B cells as previously described [66]. The ChIPmentation protocol for ≈10^6^ Hep3B cells is largely based on the protocol published by Schmidl *et al*. [67]. Details of both procedures are provided in the supplementary methods. Scaling factors were calculated from spike-in counts between samples or from sequencing depth for CTCF ChIP and applied when converting the filtered BAM files to bedgraphs with bedtools [68]. Differential analysis was performed between two datasets using the DESeq2 package [69]. To identify differential peaks rather than differential gene expression, peak files for each repeat/sample were merged into a single file in which reads were counted with featureCounts and differentially assessed with DESeq2 [70]. A cut-off threshold of FDR ≤ 0.05 was used to define statistical significance.

## Results

### Development of nucleotide resolution 4sU-seq methods: size-fractionated 4sU-Seq and single-nucleotide 4sU-Seq

**Figure 1.**
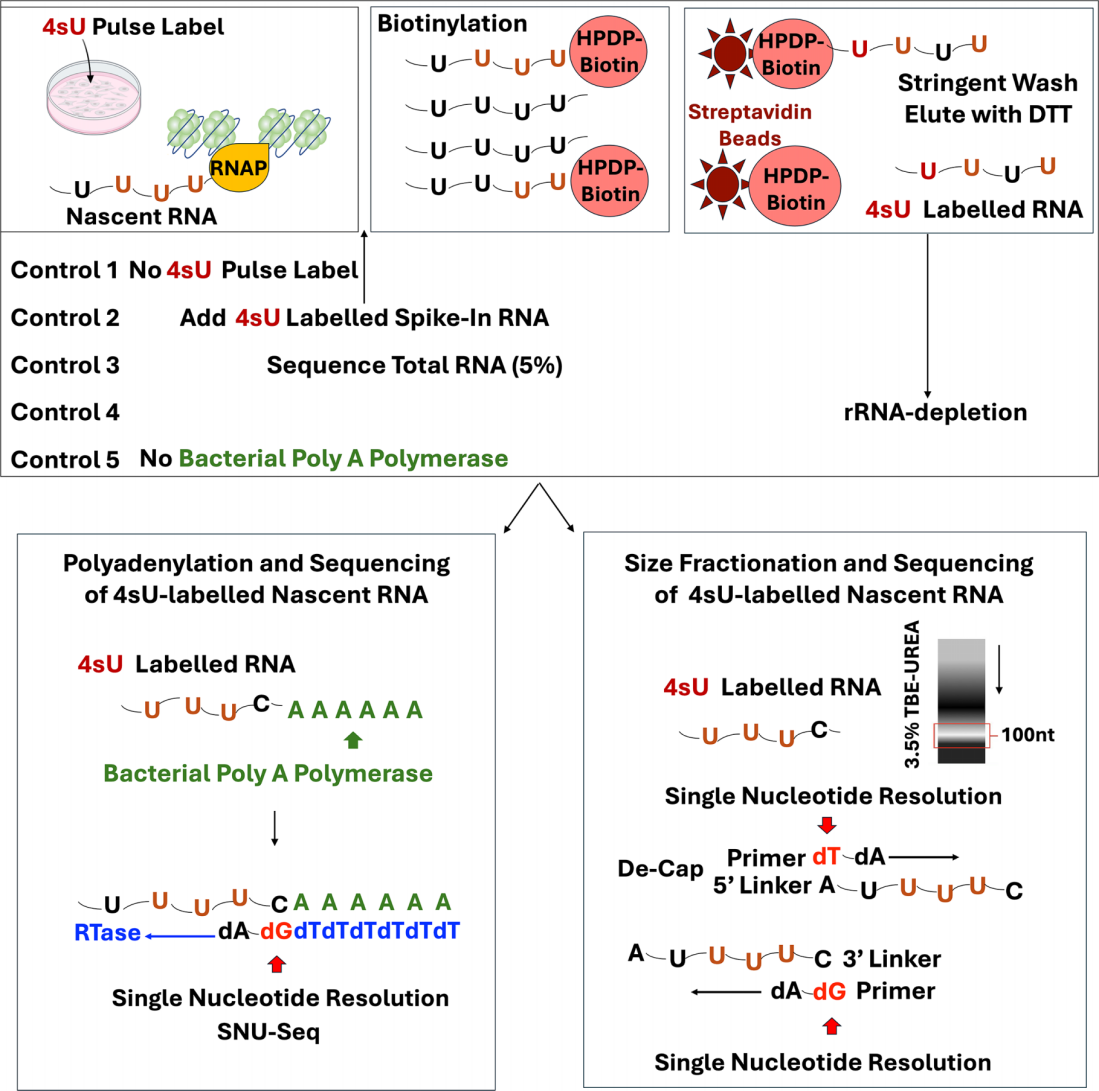
Key steps in SNU-Seq and size-fractionated (sf) 4sU-Seq. Schematic workflow for SNU-Seq and sf4sU-Seq, including thio-labelling, biotinylation, and streptavidin purification of 4sU-labelled transcripts, with controls and library construction indicating nucleotide resolution.

The aim of this work was to use the sensitivity of a 4sU pulse-label to produce two nucleotide resolution transcriptomes, known as sf4sU-Seq and single-nucleotide resolution 4sU sequencing (SNU-Seq) in adherent human cells, together with controls to enable the precise source of each read to be determined (Fig. 1 and Supplementary Fig. S1).

Optimal labelling times, reproducibility and genomic alignment were confirmed by performing TT-Seq in HEK293, HeLa, and Hep3B cells (Supplementary Fig. S2A–F). Labelling time was optimized to enable cells to process the 4sU metabolic label into a triphosphate for incorporation into transcripts, but short enough to capture primary, as opposed to processed transcripts. This is illustrated over the transcribed region of *KAZALD1*, the abundant non-coding transcription upstream and antisense to *KAZALD1* (Supplementary Fig. S2B) and around *CERS6* (Supplementary Fig. S2C), with even signal across both introns and exons.

**Figure 2.**
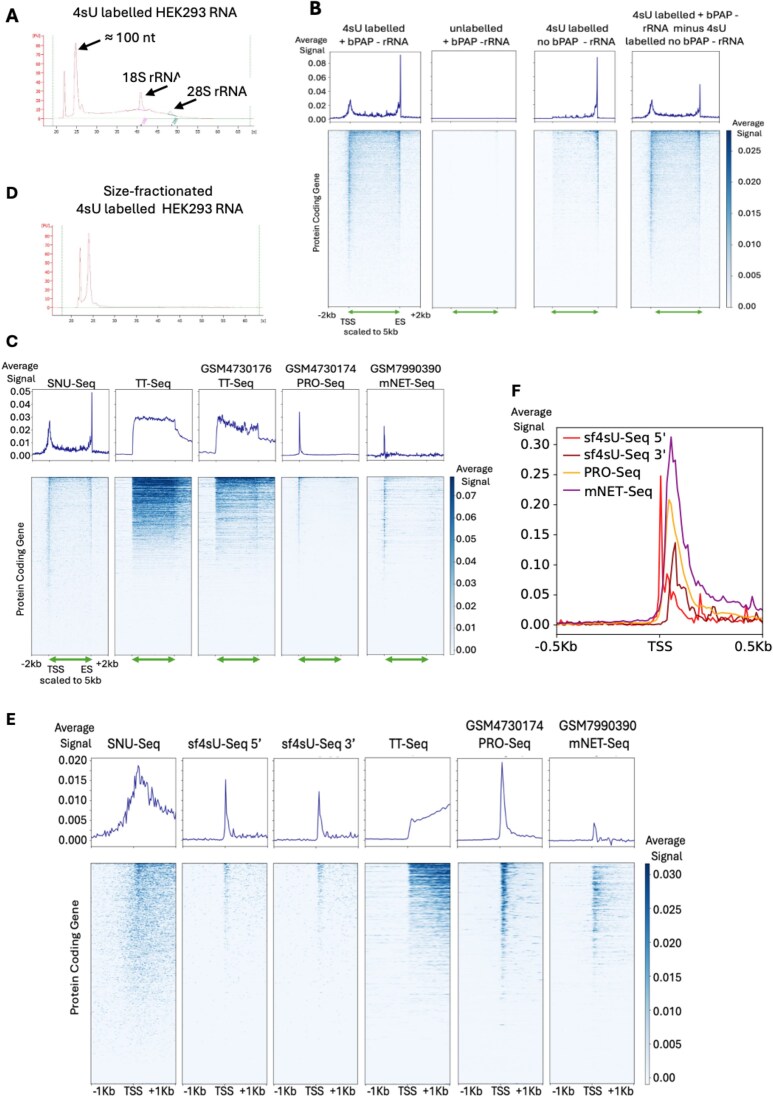
Development of single-nucleotide resolution 4sU-Seq (SNU-Seq) and size-fractionated (sf) 4sU-Seq. (**A**) Bioanalyzer RNA Pico traces of 4sU-labelled HEK293 RNA after streptavidin purification but before rRNA depletion. (**B**) Metagene profiles and heatmaps for the transcription start site (TSS) and annotated end site (ES) of 3883 GENCODE protein coding genes after blacklist removed, genomic (A residue) and outlier filtering, scaled to 5 kb (double-headed arrow), and the flanking 2 kb up and downstream shown as average normalized reads for 4sU-labelled or unlabelled samples after biotinylation and selection, rRNA depletion and treatment with or without bacterial poly (A) polymerase (bPAP). (**C**) Metagenes and heatmaps comparing SNU-Seq with TT-Seq, PRO-Seq and mNET-Seq (sources indicated) in HEK293 cells, displayed as in panel (B). (**D**) Bioanalyzer RNA Pico traces of size-fractionated (≈ 100 nt) 4sU-labelled transcripts using 3.5% TBE-urea gels. (**E**) Metagene profiles and heatmaps for 1 kb up and downstream of the TSS of 2394 GENCODE protein coding genes after blacklist, genomic (A) and outlier filtering signal shown as average normalized reads for SNU-Seq, TT-Seq (this study), PRO-Seq and mNET-Seq (sources indicated), sf4sU-Seq 5′ end and sf4sU-Seq 3′ end. (**F**) Overlay of normalized metagenes of sf4sU-Seq 5′ end and 3′ end, 0.5 kb up and downstream of the TSS (*n* = 2 394) compared to PRO-Seq and mNET-Seq (see E) (see also Supplementary Figs S1 and S2).

To generate the single-nucleotide outputs, the 4sU-labelled transcripts from HEK293 cells were processed with additional controls. First, the integrity of the 4sU-labelled transcripts was assessed after biotinylation, selection, washing and elution. An RNA pico-chip bioanalyzer trace revealed the widely distributed higher molecular weight labelled transcripts and a marked peak around 50–100 nt (Fig. 2A).

There is also a small amount of labelled rRNA in the 4sU selected material, consistent with the poor labelling of rRNA during a 10 min 4sU pulse label due to its high stability and slow processing kinetics compared to pre-mRNA [71]. The 4sU labelled and selected material was first subjected to SNU-Seq and then sf4sU-Seq.

In SNU-Seq, a poly(A) tail is added to the 3′ end of the 4sU labelled transcripts using bacterial poly(A) polymerase (bPAP), and after library preparation and sequencing, the first base next to the newly added poly(A) tail is reported (Fig. 1) [48]. For HEK293 cells, approximately 65% of reads uniquely align, and biological repeats correlate well (Pearson correlation coefficient = 0.921; Supplementary Fig. S2D). Similar read coverage is observed in both HEK293 and Hep3B cells (Supplementary Fig. S2E), with no major batch effects observed (Supplementary Fig. S2F).

The SNU-Seq outputs are displayed as normalized, scaled metagenes, and heatmaps with controls (Fig. 2B and Supplementary Fig. S3). In SNU-Seq [48] and related techniques [49], reads also arise from the host cell poly(A) polymerase (hPAP) adenylating 4sU labelled transcripts during the labelling window at PASs, consistent with the predominant signal at the 3′ region, which maps to the transcript end site (ES). Subtraction of the signal at each base for each gene in the 4sU-labelled no-bPAP samples (host PAP only) from the SNU-Seq (4sU labelled + bPAP) samples (action of both host and bacterial PAP) reveals the bPAP-dependent signal in SNU-Seq (Fig. 2B and Supplementary Fig. S3B). This leaves peaks primarily in the promoter-proximal region and around the transcript end site (ES). We note (i) that rRNA depletion improves the resolution of the signal, particularly at the 5′ region of genes, (ii) that even after subtraction of the signal resulting from the action of the host PAP, a signal remains at the ES supporting paused RNA Pol II before polyadenylation at the 3′ region, and (iii) that the SNU-Seq signal is distributed around the TSS. Evidence of transcription before the annotated transcription start site is present in IGV plots of individual genes, for example *MYC, DDIT4*, and *COX20* (see Fig. 5). After applying controls, the SNU-Seq output is compared to TT-Seq (this study and [72], PRO-Seq [72], and mNET-Seq [55], illustrating the distinct features associated with these different techniques, reviewed in [10], particularly the 5′ peak in SNU-Seq, PRO-Seq and mNET-Seq, which correlates with the PPP in metagenes (Fig. 2C) or at *CERS6* (Supplementary Fig. S3C).

sf4sU-Seq was used to investigate the origin of the abundant ≈100 nt 4sU-labelled transcripts (Figs 1 and 2A). Transcripts were eluted from a 3.5% TBE-8M Urea polyacrylamide gel (Supplementary Fig. S4A and B), their integrity confirmed on the RNA pico-chip bioanalyzer (Fig. 2D), subject to linker ligation at the 3′ end, and at the 5′ end after decapping with RNA 5′ pyrophosphohydrolase, then sequenced from both ends (Supplementary Fig. S4C). The coverage showed >86% of the library aligned to the human genome (Supplementary Fig. S4D), and after processing, it allows the first (5′) and last (3′) nucleotides of the gel-purified short transcripts to be reported (see Fig. 1). Remarkably, these short transcripts are largely derived from the promoter-proximal region of Pol II-transcribed genes, evident in metagenes and scaled heatmaps with read spikes mapping to the annotated TSS (sf4sU-Seq 5′) and about 100 nt downstream (sf4sU-Seq 3′) (Fig. 2E and F). This raises the possibility that these short transcripts are derived from RNA Pol II initiated and then paused in the promoter-proximal region (PPP), particularly as the sf4sU-Seq 3′ reads overlap with the peaks in PRO-Seq [72] and mNET-Seq [55] (Fig. 2F).

### Characterization of the promoter-proximal pause

**Figure 3.**
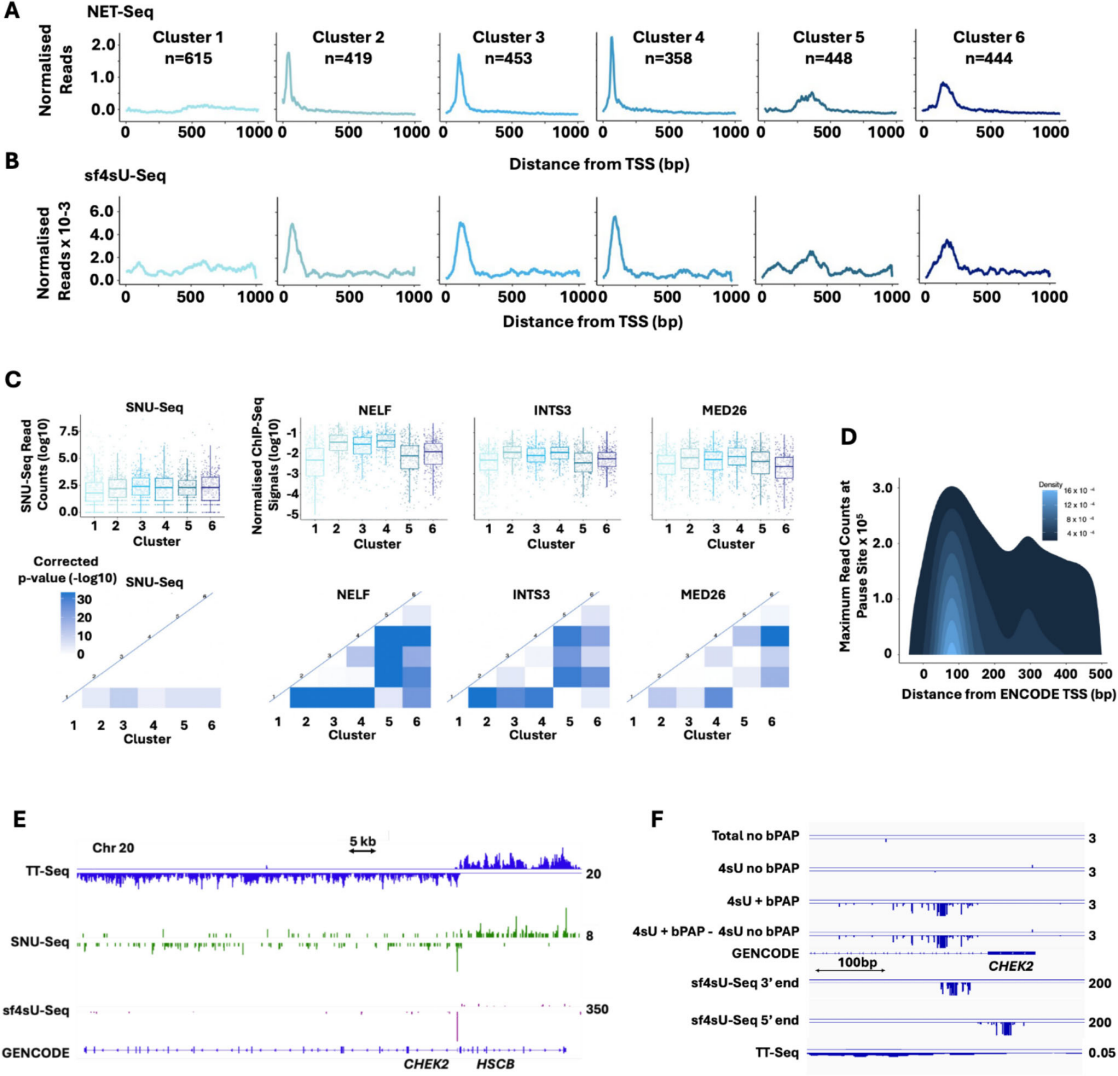
sf4sU-Seq captures the position of the PPP. (**A**) Metagenes displaying normalized mNET-Seq reads from HeLa cells over the first 1 kb of genes (scaled) for 6 clusters from the unsupervised clustering algorithm *k*-means [28]. (**B**) The gene clusters from panel (A) were used to sort normalized sf4sU-Seq 3′ end output from HEK293 cells to plot sf4sU-Seq metagenes for each cluster. Metagenes were displayed as in panel (A). (**C**) Normalized read counts from SNU-Seq and ChIP-Seq of NELF, integrator, and mediator occupancies within gene clusters 1–6. (Top) Boxplot diagrams for NELF, INTS3, MED26, and SNU-Seq levels at the 5′ end of genes for each cluster (300 bp window for ChIP-Seq and 1000 bp window for SNU-Seq). All data were scaled to the window size to ensure each gene contributes equally. All data were log_10_-transformed. (Bottom) Triangular heatmaps showing the significance of the difference between clusters for NELF, INTS3, MED26, and SNU-Seq levels. *P*-values were calculated using the Wilcoxon-rank sum test and corrected using the Bonferroni method. (**D**) Two-dimensional density plot showing the distribution of the maximum read counts from the sf4sU-Seq 3′ end library relative to the ENCODE transcription start site (TSS). Density refers to the number of occurrences per pixel (*n* = 19 874). (**E**) IGV screenshot displaying TT-Seq (HEK293, this study), SNU-Seq (HEK293, this study), and sf4sU-Seq 3′ end (HEK293, this study) profile around the *CHEK2* locus on chromosome 20. Scales are chosen to highlight read spikes. (**F**) Zoomed in view at the 5′ region of *CHEK2* for SNU-Seq signal and its controls demonstrating no polyadenylation by the host cells at the promoter-proximal region, and for the output from sf4sU-Seq at the 5′ and 3′ ends of the isolated fragments. TT-Seq indicates position of the TSS. Scales are chosen to highlight clusters (see also Supplementary Fig. S4).

Pol II accumulates at the PPP site when cells are treated with 5,6-dichloro-1-β-d-ribofuranosylbenzimidazole (DRB), an inhibitor of the Cdk9 kinase in pTEFb required for release of paused PolII into productive elongation [73]. DRB treatment of HeLa cells for 5, 10, and 15 min enabled clear stratification of genes into two classes: those without a PPP and those with a PPP, with the latter increasing with time of DRB treatment (Supplementary Fig. S4E). *k*-means clustering of mNET-Seq data, an unsupervised machine learning approach applied on the profile shape over the first 1000 nt, was used to extract distinct shape clusters from the data at all timepoints. Metagenes from a second mNET-Seq dataset [28] were optimally stratified into six clusters (*k* = 6) based on elbow and silhouette analyses (Fig. 3A). The sf4sU-Seq 3′ data were stratified into the same six clusters, and the clusters from both were plotted as metagenes (Fig. 3B). This yielded one cluster with a flat metagene, the no promoter-proximal peak class (cluster 1), and five clusters with sharp promoter-proximal peaks (clusters 2, 3, and 4) or slightly broader peaks (clusters 5 and 6) downstream of the TSS. Importantly, there was no difference in the levels of transcription, assessed using SNU-Seq, contributing to the shape of the clusters (Fig. 3C). Remarkably, the profiles, particularly the position of the peak relative to the TSS, are similar in the mNET-Seq and sf4sU-Seq 3′ library outputs. No promoter-proximal peak is evident in the same six sets of gene clusters using the TT-Seq output (Supplementary Fig. S4F–H).

Factors required for pausing, such as the NELF [74], are also expected to be enriched at the PPP. ChIP-Seq data for the NELF pausing factor [74] and the INTS3 integrator subunit [54] linked to the pausing signal by premature termination, and MED26 [75, 76] with a role in recruitment of the super elongation complex (SEC containing pTEFb) to genes destined for polyadenylation [53, 77–80] was used to assess enrichment in the chromatin at the first 1000 nt within the six clusters (Supplementary Fig. S4I–K). Metagenes of all three factors reflect those for mNET-Seq and sf4sU-Seq 3′ reads. Only the mediator ChIP-Seq profile (MED26) shows the dual peaks in cluster 5, possibly resulting from pausing at distinct sites downstream from two major but alternative TSS. This is consistent with the density distribution of 3′ end reads from sf4sU-Seq; highest around 60–80 bp downstream of the TSS (centred around 63 nts), but with a minor peak around 300 nt downstream of the TSS (Fig. 3D). That these double peaks represent pauses downstream of unannotated transcription start sites was confirmed using CoPRO, a nuclear-run on variation of PRO-Seq that enriches for promoter-proximal nascent RNAs [81], and ATAC-Seq data from HEK293 cells [82], which revealed that the additional peak in cluster five is shifted significantly further downstream compared to the other clusters (adjusted *P*-value < 3.5 × 10^–5^) (Supplementary Fig. S4L and M). The distribution of NELF, INTS3, and MED26 levels over the first 300 nt was assessed (Fig. 3C). NELF shows the greatest enrichment in clusters 2, 3, and 4, compared to INTS3 and MED26. This suggests that levels of NELF are the best predictor of a PPP, and that the majority of the 3′ end reads in the sf4sU-Seq data mark its position, as expected. A simple machine learning algorithm (logistic regression) was used to assess whether NELF, integrator, or mediator levels alone are predictive of which cluster (*k* = 4) a gene belongs to (Supplementary Fig. S5). NELF shows the strongest predictive power, compared to integrator or mediator subunits, for classifying mNET-Seq-based clusters as likely to exhibit a spike in the region of the PPP or not. As INTS3 also shows enrichment, some of the 3′ end signals in the sf4sU-Seq could arise from integrator-dependent early termination of transcription [51, 83].

At individual genes, for example *CHEK2*, a promoter-proximal peak is evident in the SNU-Seq and the 3′ end sf4sU-Seq data, but not in the TT-Seq data (Fig. 3E). Focusing on the promoter-proximal region reveals that the 3′ ends of sf4sU-Seq libraries overlap with the signal in the SNU-Seq readout at the pause site, although differences in the precise positions of the peak reads are likely to reflect differences in library preparation (Fig. 3F). The SNU-Seq control (4sU no bPAP) confirms that the promoter-proximal short transcripts are generally not subject to polyadenylation by host poly A polymerase, as there is very little signal in the 4sU labelled sample not treated with bPAP (see Fig. 2B). Taken together, these data support PolII being paused at the PPP with a templated nucleotide in its active site at many genes. However, there is evidence for the action of a host poly(A) polymerase on short divergent transcripts from some promoters such as *ZNRF3* (Supplementary Fig. S4N). Here, both divergent short transcripts, including the PDAT/PROMPT [84], have a signal in the control (4sU no bPAP), suggesting polyadenylation of their 3′ ends by hPAP.

### Using size-fractionated 4sU-Seq to discover unannotated TSS

**Figure 4.**
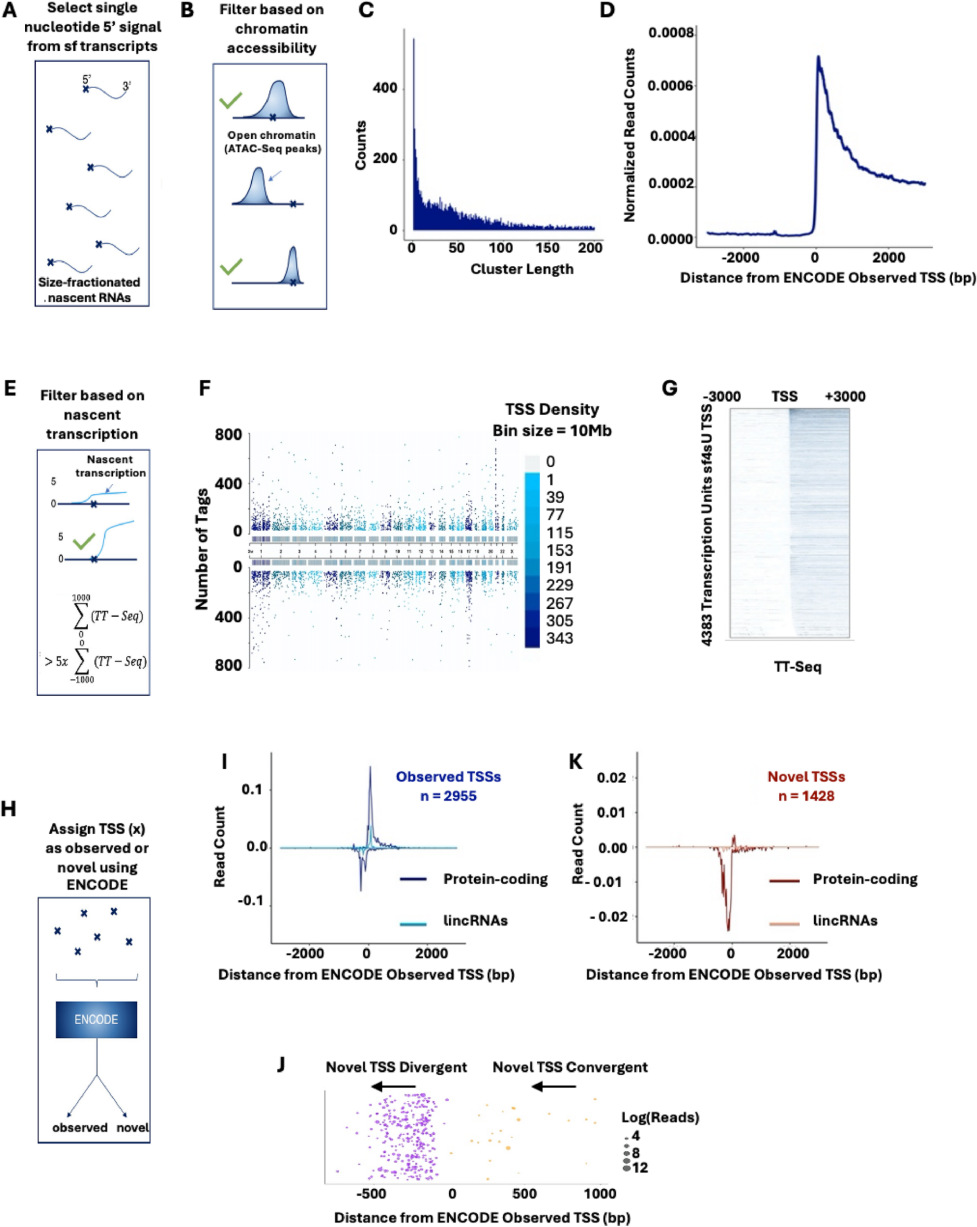
sf4sU-Seq 5′ end-based transcription start site (TSS) characterization. (**A, B, E**, and **H**) A simplified overview of the steps involved in the TSS annotation workflow linked to the data output. 5′ end signals of sf4sU-Seq were filtered through ATAC-Seq peaks and, after clustering into TSS-clusters, filtered based on a minimum 5-fold increase in the TT-Seq signal immediately downstream of the TSS candidate. The verified TSSs were then classified as either previously observed/annotated (obsTSSs) or novel/unannotated TSSs (uTSSs). (**C**) Histogram of the lengths (bp) of sf4sU-based TSS candidate clusters. (**D**) A metagene of the full-length clusters around ENCODE-annotated TSSs. The signal was scaled so that each locus contributed equally to the metagene. (**F**) Manhattan plot of the sf4sU-Seq-based TSS annotations, showing their position, including strand (+ or −) and chromosome, and quantity per position (sf4sU-Seq 5′ end signal counts on the *y*-axis) with the size of bins as the density. (**G**) Heatmap of TT-Seq data around ±3 kb of the sf4sU-Seq-based TSS annotations (signal range 0–500 reads) in descending order of the signal intensity. (**I**) Metagenes of annotated TSSs (obsTSSs, *n* = 2955) around protein-coding or lincRNA ENCODE TSSs. (**J**) Categories of the sf4sU-Seq-based TSSs that have not been annotated previously (novel/unannotated TSSs) were classified as either convergent or divergent if they are in the vicinity of annotated TSSs and on the opposite strand. The size of the data points corresponds to the log-transformed read count. (**K**) Metagenes of novel/unannotated TSSs (uTSSs, *n* = 1428) around protein-coding or lincRNA ENCODE TSSs.

The positions of read spikes in the sf4sU-Seq 5′ library align with the TSS in the metagene (see Fig. 2F). We used the data from the sf4sU-Seq 5′ library to develop a pipeline to discover new unannotated TSS and their associated transcription units (TUs) (Fig. 4), using the same principle as for START-Seq annotations [85–87].

First, only signals overlapping with ATAC-Seq peaks in HEK293 cells were used [82] (Fig. 4A and B). Second, the Paraclu TSS-clustering algorithm was used to group closely positioned TSS, as used in CAGE-Seq [88–90]. The sf4sU-Seq-based clusters were predominantly shorter than 10 bp (Fig. 4C) and cluster centres map to ENCODE TSS annotations as expected (Fig. 4D). TSS candidates for which the TT-Seq signal in the first 1000 bp downstream of the TSS candidate was lower than 5 times the signal of the 1000 bp preceding the TSS candidate were filtered out (Fig. 4E). This resulted in 4383 candidate TSSs distributed widely over the genome (Fig. 4F) and associated with active TUs mapped using TT-Seq (Fig. 4G). Finally, TSSs were assigned as previously annotated (observed TSSs or obsTSSs) or unknown/novel/unannotated TSSs (uTSSs) using the open-source code TSScall [87] (Fig. 4H). 2955 of the 4383 TSSs and associated TUs fall into the observed class, mapping around ENCODE TSSs (Fig. 4I). 98.5% of the remaining 1 428 TSSs and related TUs are associated with newly identified TSSs, and they are predominantly divergent (antisense TSS, associated with PDATs or PROMPTS [91]) and are located between 100 and 500 bp upstream of the sense TSSs (Fig. 4J), and shown in metagenes aligned to the ENCODE TSS (Fig. 4K). In conclusion, sf4sU-Seq is a viable method for transcription start site annotation and discovery, here relying on a strict set of filters, such as ATAC-Seq peaks and a 5-fold increase in TT-Seq signal, resulting in a relatively small set of TSSs. PDATs and PROMPTs are generally characterized by the depletion of the machinery containing exosomes that normally facilitate their rapid turnover [92]. The 4sU-based approaches used here have the sensitivity to detect non-coding and antisense TUs in native cells.

### Mapping alternative polyadenylation sites (APA) at the 3′ end of transcription units

**Figure 5.**
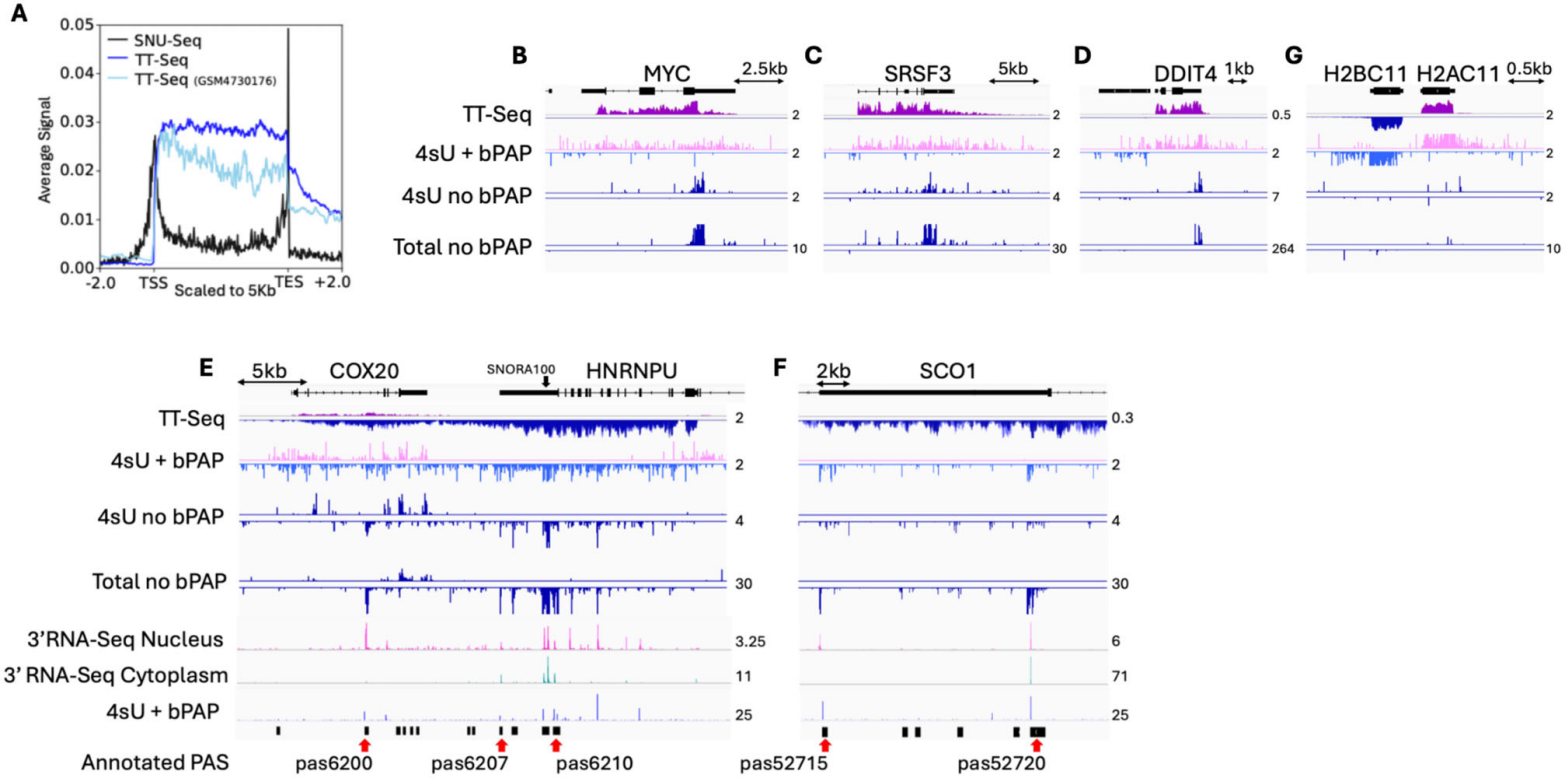
SNU-Seq detects nascent PASs. (**A**) Metagene profiles for the transcription start site (TSS) and annotated end site (TES) of 2 394 separated GENCODE protein coding genes after blacklist, genomic (A base) and outlier filtering, scaled to 5 kb, and the flanking 2 kb up and downstream shown as average normalized reads for SNU-Seq and two TT-Seq profiles (source indicated). (**B–G**) IGV snapshots showing the SNU-Seq output (4sU + bPAP) and its control for host PAP action (4sU no bPAP) in comparison to the steady-state RNA profile (Total no bPAP), and the TT-Seq profile (HEK293, this study) for (B) *MYC*, (C) *SRSF3*, (D) *DDIT4*, (E) *HNRNPU*, (F) *SCO1*, and (G) *H2BC11/H2AC11*. (**E** and **F**) Mapping nuclear (3′ end RNA-Seq) and cytoplasmic (3′ end RNA-Seq) PASs at *HNRNPU* and *SCO1* (n=3) [93], compared to the SNU-Seq output (4sU + bPAP, purple, bottom) shown at a higher *y*-axis scale. Annotated PAS [94] (boxes) on the negative strand are indicated with arrows. Scales are indicated for comparison. Numbers in each panel indicate data range, e.g. 2 refers to −2 to 0 and/or 0 to +2. *X*-axis scales are indicated in kb.

The signal at the 3′ end of transcription units in SNU-Seq was compared with the metagenes and heatmaps generated using TT-Seq (this study and [72]), PRO-Seq [72], and mNET-Seq [55] in HEK293 cells (Fig. 5A). The spike at the transcription end site (ES) in SNU-Seq overlaps with the reduction in read density in the TT-Seq output, proposed to coincide with the site of polyadenylation [39]. IGV screenshots of SNU-Seq profiles with controls at six genes were used to illustrate two features evident at 3′ regions (Fig. 5B–G). First, increased read density and spikes in the SNU-Seq (4sU + bPAP) overlap with mapped poly(A) site (PAS or pas) evident in the SNU-Seq control (4sU no bPAP, indicative of the action of host PAP during the labelling window) and in the total RNA (Total no bPAP), illustrated at *MYC* (Fig. 5B), *SRSF3* (Fig. 5C) and *DDIT4* (Fig. 5D). Second, the SNU-Seq readout (4sU + bPAP) often extends way beyond the main PAS used in the steady-state mRNA (Total RNA no bPAP), illustrated at *MYC* and *SRSF3* (Fig. 5B and C) and at some genes, this reflects cleavage and polyadenylation at alternative annotated PAS often located far downstream. This is illustrated at *HNRNPU* where the most distal PAS (pas6200) is located > 10 kb downstream of the major proximal PAS sites (pas6209/6210) for the cytoplasmically located transcripts (Fig. 5E). Separation of RNA into nuclear and cytoplasmic fractions [94, 95] reveals overlap between the 3′ end RNA-Seq reads in the nucleus and the spikes in the SNU-Seq profile, for example, at pas6200. Indeed, SNU-Seq reads together with nuclear 3′ reads spikes are evident at most of the annotated PAS, but cytoplasmic 3′ reads spikes accumulated predominantly at the proximal PASs. Thus, the profile of cytoplasmic reads is markedly different, often reflecting preferential accumulation of transcripts using the proximal PAS. SNU-Seq reads together with nuclear 3′ reads spikes are evident at most of the annotated PAS, but cytoplasmic 3′ reads spikes accumulated predominantly at the proximal PASs. This supports differential nuclear retention or export coupled to turnover of many of the nascent transcripts. The most common observation is illustrated at the *SCO1* 3′UTR (Fig. 5F). Here, there is a ≈ 2-fold difference in usage of the proximal (pas 52 720) and distal (pas52715) PASs based on the SNU-Seq readout, but the cytoplasmic signal is >10× greater, again supporting differential processing/stability of the primary transcripts in the cytoplasm (Fig. 5F). Finally, no signal in the control (4sU no bPAP; indicative of the action of the host poly(A) polymerase) is evident at histone genes (*H2BC11, H2AC11*) whose 3′ ends are not subject to host polyadenylation (Fig. 5G). Thus, the SNU-Seq output also contains information about the usage of PASs, including APA sites during the 4sU pulse labelling window.

### Determination of transcription parameters using SNU-Seq

**Figure 6.**
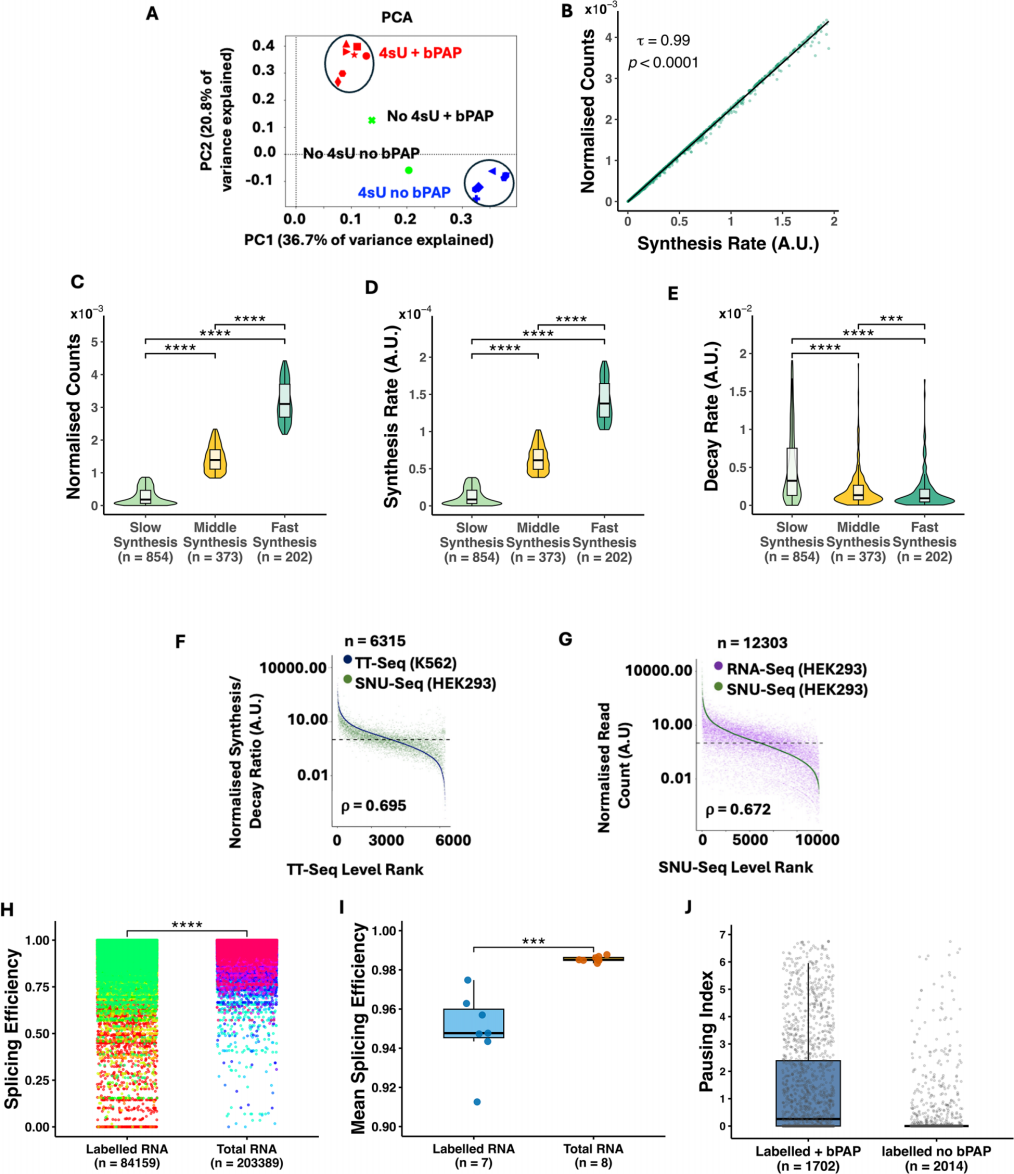
Transcription parameters derived from SNU-Seq data. (**A**) Principal component analysis (PCA) of SNU-Seq in HEK293 with six/seven repeats for 4sU-labelled samples or unlabelled control samples treated with or without bPAP as indicated. The first (*x*-axis) and second (*y*-axis) principal components (PCs) are plotted against each other in a biplot. (**B**) Correlation between synthesis rate and spike-in normalized counts, with Kendall’s tau correlation coefficient (τ = 0.99) and *P*-value (*P* < 0.0001). (**C–E**) Distributions of synthesis rate, decay rate, and spike-in normalized counts grouped by synthesis rate clusters (*k*-means = 3). The 1027 active genes are distributed across three transcriptional activity clusters: Slow Synthesis (*n* = 408), Middle Synthesis (*n* = 403), and Fast Synthesis (*n* = 216). The significance of differences between clusters was assessed using the Wilcoxon test; ^****^P < 0.0001, ***P < 0.001. (**F**) Ranked correlation plot of the synthesis-to-decay ratios between SNU-Seq (this study) and the original TT-Seq dataset in K562 cells [39]. The Spearman correlation coefficient (rho = 0.695) is indicated. (**G**) Correlation between SNU-Seq (this study) and published RNA-Seq in HEK293 cells [61]. The Spearman correlation coefficient (rho = 0.672) is indicated. (**H–I**) Splicing efficiency and mean splicing efficiency derived for total RNA preparation (Total no bPAP; n = 7) compared to SNU-Seq (labelled RNA, 4sU-labelled + bPAP; *n* = 6) in HEK293 cells. ^****^P < 0.0001, ***P < 0.001. (**J**) Pausing index comparing 4sU-labelled RNA treated with bPAP (SNU-Seq) or without bPAP (host PAP control).

Six repeats of the SNU-Seq (4sU + bPAP) output compared to the controls processed without bPAP treatment, and showing good reproducibility (Fig. 6A), were used to explore transcription parameters in HEK293 cells. The synthesis rate (SR) and decay rate (DR) were calculated for 2 394 genes in the annotation subset after thresholding and removing reads in ENCODE blacklisted regions: DR = −(1/time) × log(1 − RNA_4sU_/RNA_total_), and SR = RNA_total_ × DR, where time is 10 min, RNA_4sU_ is the normalized SNU-Seq counts (4sU + bPAP), RNA_total_ is the normalized total RNA counts (total no bPAP), exploiting the 4sU-labelled nascent and steady-state total RNA signal from the same samples [39, 60]. The density of reads over the gene body directly relates to their synthesis rates (Kendall’s τ = 0.99) (Fig. 6B). Based on an elbow analysis of the calculated synthesis rates, *k*-means clustering (*k* = 3) was applied to classify genes into fast, middle and slow groups (Fig. 6C). In each group, fast synthesis rates have higher spike-in normalized counts (Fig. 6C and D) and lower decay rates (Fig. 6E), like parameters determined in *S. cerevisiae* [96]. Finally, the transcription elongation profiles at 6315 genes determined using SNU-Seq are broadly similar to those generated using the TT-Seq protocol in K562 cells [39, 60], with a Spearman correlation of 0.695 (Fig. 6F).

### Determination of splicing efficiency

Comparison of the SNU-Seq output with mature transcript levels at 12 303 genes in HEK293 cells reveals a Spearman correlation of 0.672 (Fig. 6G). The differences in the profiles should reflect intronic transcription enriched during the labelling window in the SNU-Seq output compared to co-transcriptionally processed mature mRNA transcripts, which will also be present in the total output [97]. To assess the level of pre-mRNA processing captured during the labelling window by SNU-Seq, the primary sequence output (101 nt) obtained from the total RNA libraries (*n* = 7) and the SNU-Seq (4sU labelled RNA) libraries (*n* = 6) were used to calculate the splicing efficiency from the ratio of spliced to unspliced reads at splice junctions using SPLICE-q [62] (Fig. 6H). Then, the mean splicing efficiency (SE) score was computed by averaging all measured splicing events in the total RNA libraries and SNU-Seq libraries prepared from the same samples (Fig. 6I). The results were comparable to those obtained using a 15 min bromodeoxyuridine (BrdU) pulse-label of HEK293 cells compared to an unlabelled total RNA preparation used in the development of the SPLICE-q algorithm [62], indicating that SNU-Seq captures more transcripts with introns (pre-mRNA) in comparison to the total RNA.

### 5′ End pausing index

Metagene profiles and IGV snapshots indicate read peaks near the TSS of transcription units, likely representing promoter-proximal pausing or poor elongation kinetics of initiated RNA polymerase II. This pause is usually captured in 4sU-labelled samples when treated with bPAP, as the output from 4sU-labelled samples not treated with bPAP primarily captures reads due to polyadenylation by host PAP of pre-mRNAs undergoing 3′ end formation during the labelling window. The read counts within the TSS proximal window (−50 bp to + 200 bp) were compared to those from +200 bp to the start of the 3′ UTR for the two data sets, as previously done [98] (Fig. 6J). In the promoter-proximal region (−50 to + 200), the bPAP-treated labelled transcripts show a 1.865 median log2 fold increase in reads (≈ 3.65-fold) compared to untreated (no bPAP) samples.

To characterize the regulatory context, the calibrated pausing signal was obtained by dividing the summed sf4sU-Seq 3′ signals for each gene (50–150 bp downstream of the TSS) by the sum of TT-Seq reads for each gene (0–500 bp downstream of the TSS, Supplementary Fig. S5G). Gene ontology analysis of the top and bottom deciles of genes ranked by their relative pausing signal revealed that genes exhibiting pausing are involved in primary metabolic processes, cell-cycle regulation and DNA-damage checkpoints (Supplementary Fig. S5H). This agrees with previous analyses on promoter-proximal pausing based on Pol II ChIP-Seq [98]. Because of the single-nucleotide resolution, as well as the high sensitivity of this approach, highly statistically significant results on the gene ontology of promoter-proximal pause were generated compared to the ChIP-Seq approach [98], which generates lower-resolution Pol II positioning profiles rather than precise information about the act of transcription.

### Characterization of enhancers in Hep3B cells

**Figure 7.**
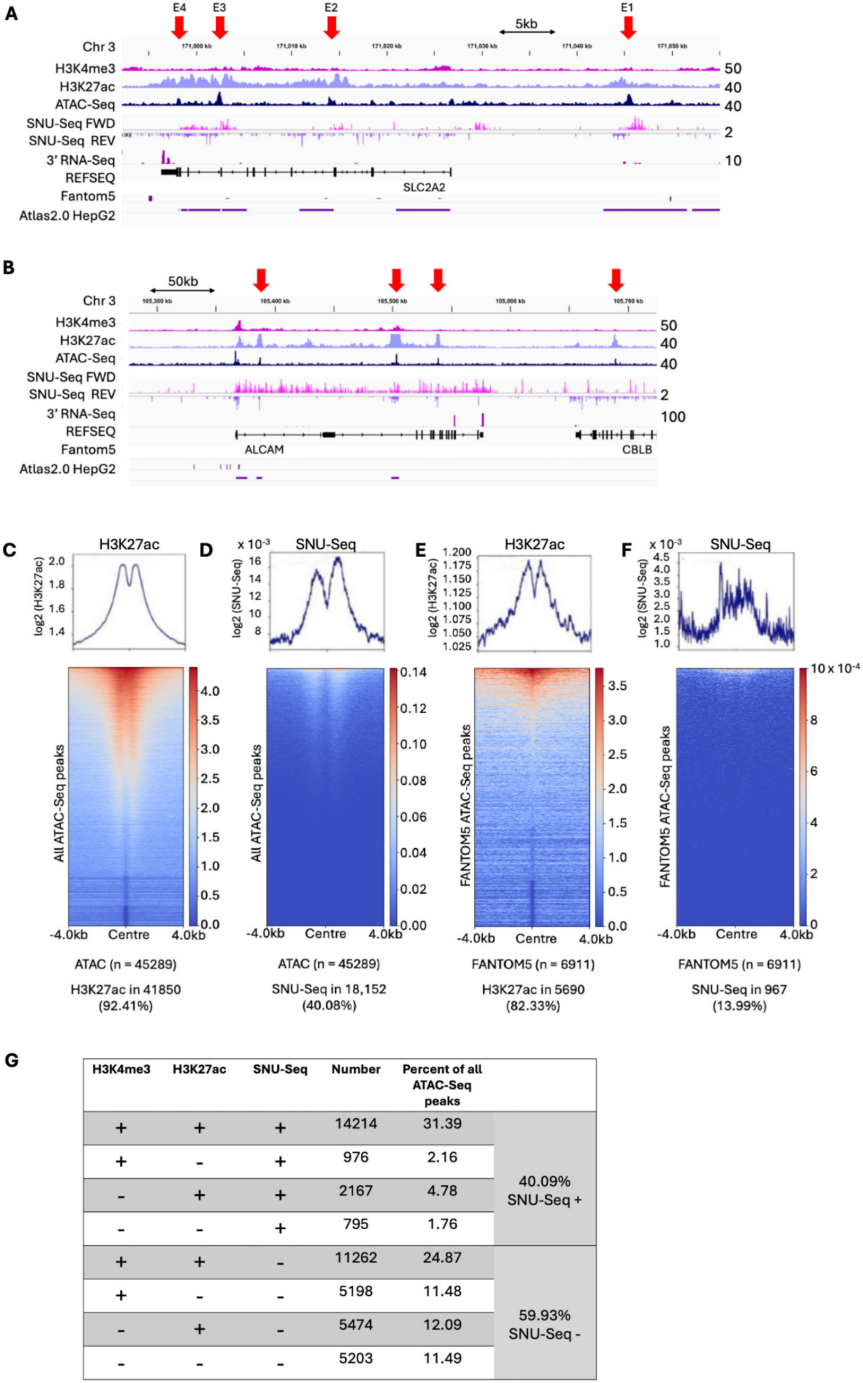
The chromatin environment and transcription in Hep3B cells. (**A** and **B**) IGV snapshots showing chromatin features and transcription around two loci selected as expressed in hepatocytes with characterized enhancers (including FANTOM5 and Hep2G Enhancer Atlas annotations) indicated by vertical arrows for (A) *SLC2A2*, E1 to E4 and (B) *ALCAM*. Numbers by each panel indicate the *y*-axis data range (e.g. 2 is −2 to 0 and/or 0 to +2 for SNU-Seq). The *x*-axis scale is indicated in kb. (**C–F**) Metagenes and heatmaps showing (C and E) H3K27ac or (D and F) SNU-Seq distribution around all (C and D) ATAC-Seq peaks (*n* = 45 289) or (E and F) FANTOM5 annotations (*n* = 6 911). (**G**) Number of chromatin features around ATAC-Seq peaks that are associated with SNU-Seq reads (+) or not (−) (see also Supplementary Figs S6 and 7).

Active enhancers are often characterized by open chromatin (ATAC-Seq) marked with H3K27ac and divergent eRNAs [99]. SNU-Seq was combined with chromatin analysis in Hep3B hepatocellular carcinoma cells, which have well-characterized enhancers [100, 101], and used to illustrate how frequently features co-occur around individual genes (Fig. 7A and B, and Supplementary Figs S6A–D) and genome-wide (Fig. 7C–F).

SNU-Seq can detect divergent eRNA transcripts at previously characterized functional hepatocyte enhancers defined by their interaction with the enhancer-looping factor LDB1, including the four enhancers E1-E4 at *SLC2A2* (Fig. 7A) and three enhancers within *ALCAM* (Fig. 7B) [101]. Divergently transcribed regions with similar characteristics are evident in three regions; around *HNF4A*, encoding a liver-specific transcription factor, at the *STAT1* enhancer and at 4 regions around *DDIT4* (Supplementary Fig. S6). Divergent transcripts extending >1 kb in each direction are evident at both the promoters and putative enhancers in and around these genes, with enhancers within the transcribed region identifiable by transcription on the antisense strand, as well as ATAC-Seq peaks often flanked by H3K27ac-marked nucleosomes. Notably, H3K4me3 at the enhancers and the super-enhancer (SE_35 550) around *DDIT4* are often associated with CpG islands and divergent transcription (Supplementary Fig. S6C). A comparison of the ability of mNET-Seq, SNU-Seq, PRO-Seq and TT-Seq to detect eRNAs at the four FANTOM5 annotated enhancers around *DDIT4* revealed marked differences in the sensitivities of each method, compared to the number of reads over the *DDIT4* gene in HEK293 cells (E, arrows; Supplementary Fig. S6D).

The genome-wide analysis was focused on ATAC-Seq peaks (this study) or FANTOM5-annotated enhancers. The datasets were filtered to retain only features on autosomal chromosomes (chr1-22) and to remove any features closer than 5 kb from any similar feature. For ATAC-Seq annotations located up to 1 kb from a GENCODE gene, only those 5 kb apart from any other ATAC-Seq peaks were kept, using this gene’s strand information. This yielded 17 367 annotations on the forward (FWD) strand and 16 570 on the reverse (REV) strand. If features were >1 kb away from a GENCODE gene, only those at least 5 kb from other peaks were kept and annotated as intergenic features (*n* = 13 434). All features, regardless of position, and at least 5 kb apart from each other, constituted the third group, *n* = 45 289 (all). FANTOM5 enhancers were filtered, retaining those at least 5 kb from any GENCODE annotated gene and 5 kb apart from each other, yielding 6911 features for analysis.

Heatmaps and metagenes of the concatenated and log_2_-transformed values for SNU-Seq, H3K27ac, and H3K4me3 were plotted at peaks of open chromatin (all ATAC-Seq peaks). The data revealed that open chromatin is often associated with nucleosomes marked with H3K27ac (Fig. 7C) and, interestingly, H3K4me3 (Supplementary Fig. S7A and B) but fewer sites have measurable SNU-Seq reads (Fig. 7D, and Supplementary Fig. S7C and D). Similar features were evident at FANTOM5 annotated enhancers (Fig. 7E and F, and Supplementary Fig. S7B).

The data were parsed so that features around each ATAC-Seq peaks could be identified (Fig. 7G) and expressed as present (+) or absent (−) after thresholding (SNU-Seq (>0 for log_2_(*x* + 1)), H3K27ac and/or H3K4me3 (>1.25 for log_2_(*x* + 1)) values to eliminate noise and reliably identify peaks or signals (Supplementary Fig. S7F). SNU-Seq reads were identified at 40% of ATAC-Seq sites while 60% had no detectable reads, despite many being associated with transcription-associated modification such as H3K27ac or H3K4me3. The failure to detect divergent transcription at so many sites of open chromatin could simply reflect a technical failure of SNU-Seq to detect transcription. However, these data also raises the interesting possibility that these regions may be primed for subsequent transcription when appropriate signals are received. This was addressed using Hep3B cells, which induce a temporal transcriptional response to IFNγ involving JAK–STAT1 signalling, activating the interferon response transcription factors (IRF), including IRF1 and IRF8 [102].

### IFNγ-responsive transcription at primed chromatin

**Figure 8.**
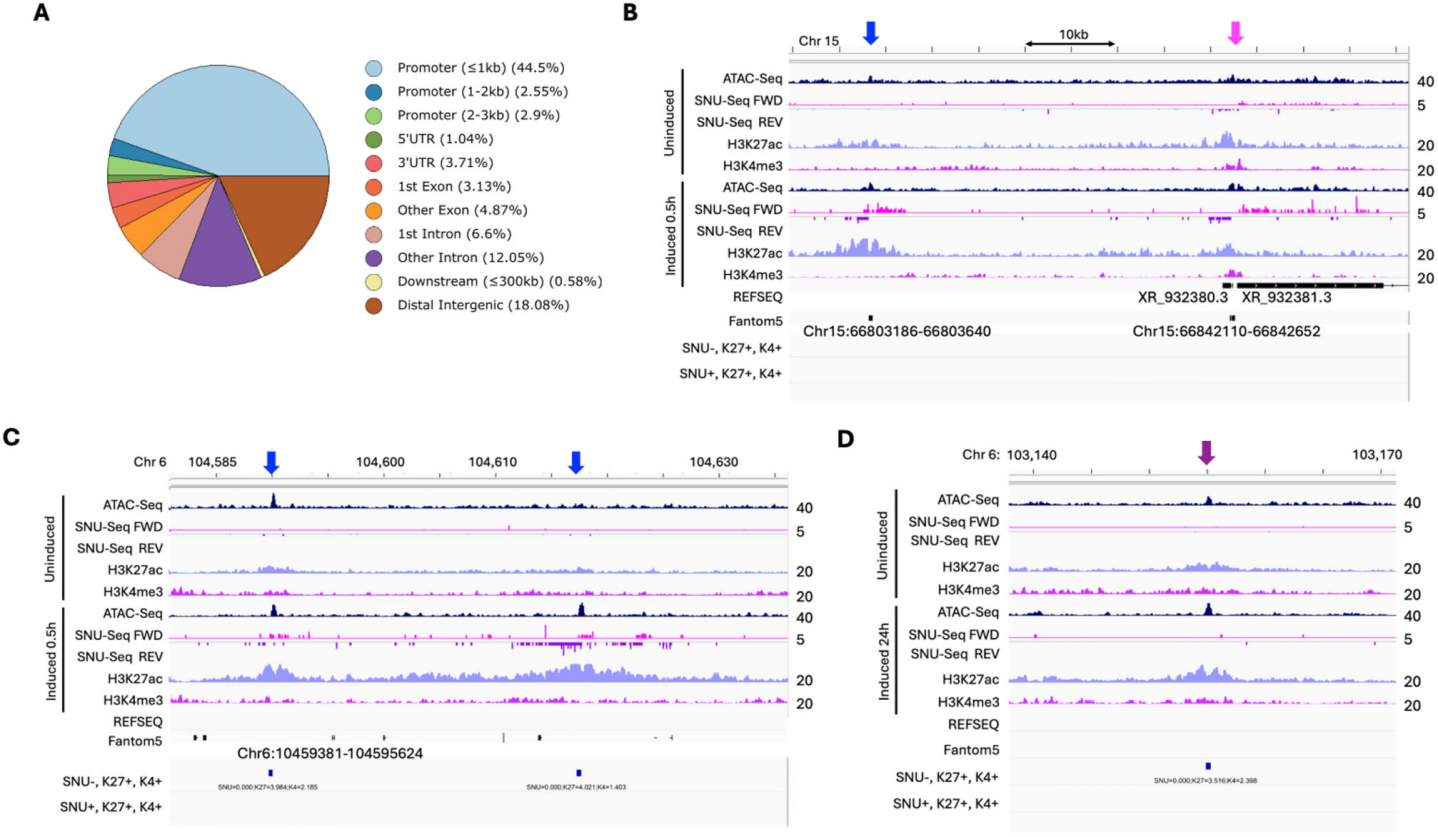
Primed sites of chromatin in Hep3B cells showing IFNγ-inducible transcription. (**A**) Genomic distribution of SNU-Seq data upon IFNγ-induced transcription for 0.5, 2, or 24 h in Hep3B cells. (**B–D**) IGV snapshots showing chromatin features (ATAC-Seq; H3K27ac; H3K4me3) and transcription (SNU-Seq) at indicated genomic regions, selected for the following features: (B) constitutive transcription (pink arrows); (B and C) transcription induced at 0.5 h (blue arrows); (D) no transcription induced by 24 h (arrow).

A genome-wide analysis revealed 635 ATAC-Seq peaks showing IFNγ-dependent increased transcription at either 0.5, 2, or 24 h post-induction (Fig. 8A). 282 regions (44.5%) are located at gene promoters based on GENCODE annotations with the remainder upstream, downstream or within genes. Examples of intergenic primed regions (SNU−, ATAC+) that induce divergent transcription after treatment with IFNγ, include a FANTOM5-annotated enhancer on chromosome 15 (Fig. 8B) and two unannotated chromosomal locations on chromosome 6 (Fig. 8C). These three regions induce transcription within 0.5h of IFNγ treatment. A third potentially primed “SNU−, ATAC+” region on chromosome 6 remains primed at all time points (arrow in Fig. 8D), suggesting this region might respond to different signals. Note that H3K27ac levels increase at 2 h induction, although there is no detectable transcription from this region.

**Figure 9.**
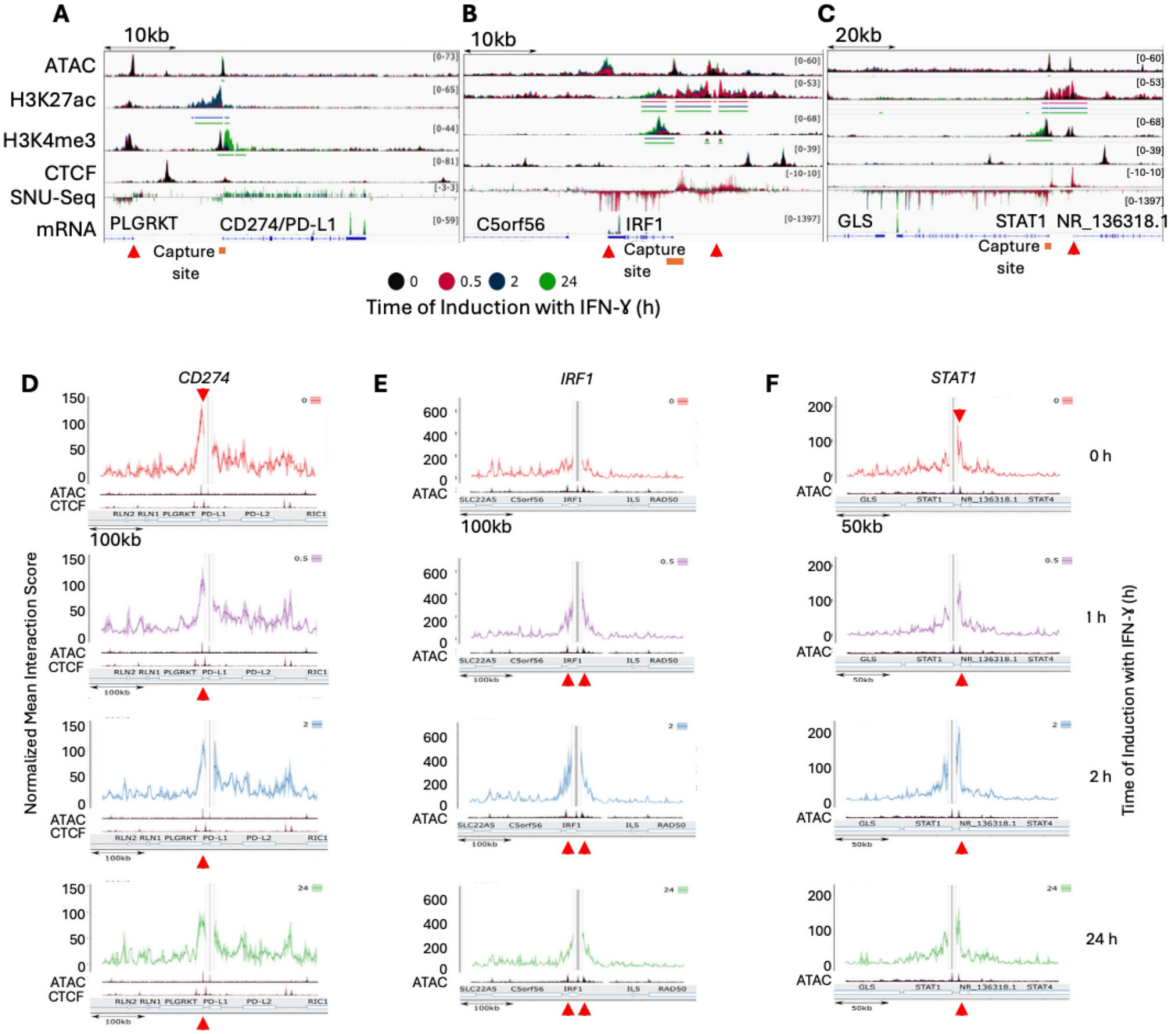
Long-range chromatin interactions at genes with IFNγ inducible transcription in Hep3B cells. (**A–C**) IGV snapshots showing overlays of chromatin features (ATAC-Seq, H3K27ac, K4me3, CTCF), position of mRNA PASs using 3′ end-Seq reads (Total no bPAP) and SNU-Seq profile (4sU labelled + bPAP) in uninduced cells (black) or after 0.5 h (pink), 2 h (blue), or 24 h (green) IFNγ induction. Supplementary Fig S8 shows separated tracks at key time points. Regions with statistically significant signal changes are indicated by pink, blue, or green lines under the H3K27ac and H3K4me3 signal, where the colours correspond to the indicated treatment time. Read count ranges are to the right of each track. Capture sites (Capture-C) are indicated with orange bars located at the promoters of (A) *CD274*, (B) *IRF1*, and (C) *STAT1*. Arrowheads indicate the position of maximum mean interaction with the promoter (exemplified in D–F). (**D**–**F**) Normalized (to *MYC*) mean interaction score (*n* = 2) at 0, 0.5, 2, and 24 h of IFNγ treatment using Capture-C anchored to the promoter of (D) *CD274*, (E) *STAT1*, or (F) *IRF1*. Reads were normalized based on the number of *cis* reads per 100 000 reads. The data were smoothed using mean read counts within a 2 kb window. ATAC-Seq peaks and CTCF binding sites peaks at equivalent times of induction ar shown. Arrowheads indicate sites of maximum interaction score at the various times before (top panel) or after induction. Scale (kb) is indicated under the uninduced locus track (see also Supplementary Fig. S8).

Primed IFNγ-responsive promoters include *CD274/PD-L1* that induces transcription at 2 h (Fig. 9A and Supplementary Fig. S8A) and *IRF1* that induces transcription at 0.5 h (Fig. 9B and Supplementary Fig. S8B). *STAT1* is expressed at low levels in uninduced cells but becomes upregulated within 0.5 h of IFNγ treatment (Fig. 9C and Supplementary Fig. S8C). Enhancers or putative enhancers for *CD274/PD-L1, IRF1*, and *STAT1* also become induced and show divergent transcription at 0.5 h (Fig. 9A–C and Supplementary Fig. S8A–C). This analysis supports the idea of a highly poised and responsive Hep3B epigenome for both putative enhancers and their promoters and at widespread intergenic regions.

### Chromatin looping at IFNγ-dependent genes

Changes in chromatin looping, as detected here using Capture-C, can provide functional validation of enhancer-promoter interactions induced in response to IFNγ treatment (Fig. 9 and Supplementary Fig. 8D–I). Capture-C is a one-to-all 3C method in which chromatin is cross-linked to proximal regions in 3D space, NlaIII-digested and ligated within intact nuclei, resulting in a DNA library containing NlaIII-digested fragments joined based on their spatial distance [103]. Biotinylated Capture-C probes (orange lines; Fig. 9A–C) were designed to capture any NlaIII restriction enzyme fragments spanning the *CD274, STAT1*, or *IRF1* target promoters, allowing any region of DNA in close spatial proximity to the region of interest to be identified. In uninduced cells, peaks of open chromatin (ATAC-Seq) are evident at the *CD274, STAT1*, or *IRF1* promoters and at their putative enhancers: *STAT1*, 5.3URR; *IRF1*, GeneHancer identifier GH05J132484; *CD274, PLGRKT* Epromoter, all located about 5–10kb upstream of the promoter [104, 105].

Capture-C against the *CD274* promoter revealed a predominant peak of interaction to the nearest gene promoter, *PLGRKT*, a plasminogen receptor protein, which is evident in the uninduced samples and does not change with IFNγ treatment over time (Fig. 9D, and Supplementary Fig. S8D and G). Each sample and repeat contains similar percentages of *cis* reads over total reads demonstrating similar library qualities. *PLGRKT* is constitutively expressed, with transcription and full mRNA transcripts of the gene identified in every sample tested. No significant changes were observed in either 3′-RNA-seq or SNU-Seq using DESeq2 despite a putative IRF1 binding site in the *PLGRKT* promoter, as well as both pSTAT1 and IRF1 binding sites at the *CD274* promoter. This raises the possibility that expression of *CD274* is dependent on elements with the characteristics of an Epromoter, commonly associated with interferon response genes [106, 107] or alternatively is associated with transcription hubs or clusters [108, 109]. Indeed, although *PLGRKT* mRNA is expressed at all time points with a low level SNU-Seq output over the gene body, the high levels of divergent transcription around the promoter region resemble an enhancer (Fig. 9A and Supplementary Fig. S8A). This suggests that promoters such as *CD274* are primed with a long-range interaction to a putative divergently transcribed Epromoter, enabling the *CD274* promoter to respond to regulated transcription factors, such as IRF1, produced between 1 and 2 h post-induction.

The predominant long-range interactions with the *IRF1* promoter are within approximately 10kb upstream of the promoter and only occur after IFNγ induction (at 0.5 h) when divergent transcription at the promoter and enhancer is evident and remain significant at 2 h (Fig. 9B and E). Interactions were evident although no longer significant by 24 h despite similar library quality (Supplementary Fig. S8E and H). No interactions can be seen in trans. These data suggest that large-scale rearrangements of the chromatin in 3D space are required at *IRF1* to bring the promoter and enhancer in proximity, coincident with divergent transcription at both the enhancer and promoter.

The pattern of long-range interactions between the *STAT1* promoter and its putative upstream enhancer (5.3URR; NR_136318.1) were similarly significantly increased at 0.5 h (compared to uninduced) when transcription is significantly induced (Supplementary Fig. S8F and I) and this interaction is maintained over 24 h (Fig. 9F). However, interactions and low levels of transcription at about 8% of the levels at 0.5 h were also evident before induction suggesting that the *STAT1* promoter is already primed for additional transcriptional induction by an interaction with its enhancer (Fig. 9C and F).

In summary, at these three loci, nascent divergent transcription at elements with characteristics of enhancers correlates with chromatin looping to nearby promoters, but not always with the onset of pre-mRNA transcription. This suggests different levels of epigenetic priming in Hep3B cells, which can be uncovered using SNU-Seq and chromatin analysis to monitor the response to IFNγ.

## Discussion

The aim of this work was to develop and validate sf4sU-Seq and SNU-Seq [48] to interrogate transcriptomes in HEK293, and in Hep3B cells before and after induction with IFNγ, with high sensitivity and high resolution, allowing the precise source of reads to be determined over pre-mRNA transcription units and at their control regions.

SNU-Seq [48] is similar to the pA+/pA− technique developed by the Jensen group [49] and used by them to characterize factors that control the degradation of non-coding transcripts [13, 16, 50, 59] based on a protocol originally developed to compare transcriptional and post-transcriptional events using 3′ end RNA-Seq in *S. cerevisiae* [41]. Here we develop sf4SU-Seq which when combined with SNU-Seq allows detailed characterization of the TSS on both native pre-mRNA transcripts and their divergent PROMPTs/PDATs, helping to define the TSS at >1500 unannotated PDATS and the position of the PPP during transcription of pre-mRNAs. This was aided by the discovery of an abundant fraction of native 4sU pulse-labelled transcripts on gels around 100 nt long. rRNAs and tRNAs that fall into this size range are not enriched in the 4sU selected transcripts due to their stability combined with the short pulse-labelling times used here. Isolation and sequencing of these short transcripts reveal that the majority map to the promoter-proximal region of divergent transcription units including pre-mRNAs and PDATs being found around 63 nt from the TSS. Interestingly, unsupervised clustering of genes, based on the shape of the NET-Seq profile over the first 1000 nt of genes, reveals some genes with no obvious DRB-sensitive PPP, while others have a sharp promoter-proximal peak that correlates with NELF enrichment, is independent of the level of transcription over the genes, and increases in intensity when release into productive elongation is blocked by DRB treatment. This contributes to the validation of the transcript peaks in the sf4sU libraries mapping to RNA polymerase II with their 3′ ends arrested at the PPP, as they fall into the same shape clusters as observed in the NET-Seq data. The PPP occurs within longer transcription units, either pre-mRNAs or PDATs which extend >1 kb from the TSS when released from the pause. This is despite the actions of complexes such as restrictor which contributes to slowing polymerase to facilitate early termination of transcription, particularly of non-coding transcripts such as PDATs and eRNAs [17], and extends the current views about short and long eRNAs [110]. One caveat of SNU-Seq is that it is not known whether all the >1 kb divergent eRNAs are produced from one contiguous transcription event, but this can be addressed using sf4sU-Seq, as gel-based isolation of the transcripts ensures that the mapped 5′ and 3′ end sites result from contiguous transcription. In mammalian genomes, transcription over promoters and between overlapping genes is common [111], but whether these arise from contiguous transcription is not clear. One disadvantage of many techniques used to assess ongoing transcription is the fragmentation of either the RNA or cDNA, and thus, it can be hard to assess whether reads arise from contiguous transcription or distinct transcription units. Moreover, these regions are excluded from this study to ensure clarity. Gel-based size fractionation of native transcripts into different size ranges before library construction will define the extent of transcription units, as has been done in *S. cerevisiae* to map extensive regulatory overlapping transcription, antisense transcription and transcriptional interference throughout the genome using sfNET-Seq [30, 112, 113]. The longer native 4sU-labelled human transcripts present on the TBE gels in this study are ideal for size fractionation. Analysis might be complicated by co-transcriptional splicing of the longer genic transcripts, expected to reduce the size of these native transcripts but could yield additional information on splice site usage [114].

SNU-Seq has many advantages compared to other methods for mapping mammalian transcriptomes. SNU-Seq retains full-length fragments followed by mapping the last incorporated nucleotide, allowing the presence of PPPs and multiple nascent PASs to be defined in the same experiment, unlike TT-Seq. mNET-Seq and PRO-Seq define the PPP, but not PAS usage. The single-nucleotide resolution of SNU-Seq produces an even, uniform signal over gene bodies representing productive elongation, with read density directly related to synthesis rate, and can be used to parameterize simulations to enable the derivation of other transcription metrics [115]. SNU-Seq is robust and reproducible, producing profiles that are similar across repeats and across different human cell lines.

SNU-Seq enabled us to map divergent transcription units throughout both the Hep3B and HEK293 genomes. This ubiquitous transcription initiates in regions of open chromatin, remodelling the local chromatin structures and associated DNA and histone modifications, and may lead to changes in higher-order chromatin structures [116], as demonstrated here for IFNγ-responsive loci. Recent high-resolution Micro Capture-C ultra (MCCu) analysis [117] of chromatin at a limited number of key loci in mESC reveals both long-range contacts between enhancers and promoters and local interactions within enhancers driven by transcription factor-mediated nucleosome depletion. These nucleosome-depleted regions are proposed to coalesce into an interchromatin compartment, enriched with RNA polymerase II, flanked by aggregated acetylated nucleosomes, driving compartmentalization adjacent to the interchromatin compartment. As our transcription data has the sensitivity to detect many divergent transcription units, we can relate functional elements such as the 30 kb super-enhancer SE_35 550 upstream of *DDIT4* and other enhancers surrounding *DDIT4* with chromatin features and transcription. In addition to divergent transcription from regions of open chromatin, flanked by H3K27ac-enriched nucleosomes, H3K4me3 enrichment is often observed, and correlates with annotated CpG islands including at many enhancers. Interestingly, super-enhancers such as SE_35 386 over *SLCA2A* and SE_35 550 upstream of *DDIT4* harbour multiple divergent transcription units but either lack CpG islands or if they do show reduced levels of H3K4me3 relative to enrichment for H3K27ac, as expected [118, 119].

In Hep3B cells, stimulation with IFNγ is associated with the formation of new higher-order structures (enhancer–promoter interactions) in chromatin. Here, we demonstrate two ways in which the genome can be primed to respond to regulatory signals, such as IFNγ. First is primed open chromatin, often flanked by H3K27ac nucleosomes, but lacking detectable transcription, present at the *IRF1* enhancer and promoter before induction with IFNγ. Here, the presence of H3K27ac at the putative enhancer and promoter may reflect an active primed state, but the onset of transcription is linked to a further change in the functional state and correlates strongly with the formation of long-range interactions between the enhancer and promoter as predicted by current models [120].

The second level of priming is the formation of constitutive chromatin loops between a divergently transcribed enhancer and transcriptionally silent promoter, exemplified at *CD274/PD-L1*. This state is maintained until the *CD274* promoter is activated at 2h post-induction, consistent with IFNγ-dependent production of IRF1 protein, required to activate the *CD274* promoter. These primed sites may provide a memory or bookmark to enable rapid transcriptional induction when conditions change, directed by activated transcription factors. At *CD274*, constitutive long-range interactions with an element resembling an Epromoter [109] with constitutive divergent transcripts are temporally distinct from the onset of IFNγ-inducible *CD274* transcription, suggesting different primed states. SNU-Seq will be instrumental in understanding how regions of the genome with open chromatin respond to new signalling pathways and in mapping changes in long-range interactions involving these regions, which correlate strongly with phenotype [121]. This is exemplified in human whole-blood chromatin, where the network of juxtaposed genomic regions changes with disease states, and can be used as a diagnostic and prognostic tool in the clinic. In these patients, anchor sites are often associated with H3K27ac [121, 122], although whether these changes in humans are a consequence of transcriptional changes or not remains to be determined, and SNU-Seq will be instrumental in this.

## Limitations of these studies

The major limitation of SNU-Seq and sf4sU-Seq and the associated data interpretation is that they both may lack the sensitivity to detect transcripts, especially from the regions described as being epigenetically primed, and acknowledge that very unstable exosome sensitive transcripts may be produced.

## Supplementary Material

gkag652_Supplemental_File

## Data Availability

The datasets generated in this study are available in the NCBI Gene Expression Omnibus (GEO) under accession numbers GSE172053 (Hep3B cells), GSE165251, and GSE179306 (HEK293 cells). Scripts for the SNU-Seq data processing and analysis are available at https://github.com/ugerlevik/SNU-seq or https://doi.org/10.5281/zenodo.19616293.
