# Supplementary material for: Nucleotide resolution 4-thiouridine sequencing by SNU-Seq and sf4sU-Seq reveals the transcriptional responsiveness of an epigenetically primed human genome
Source: Nucleic Acids Res. 2026 Jun 27;54(12):gkag652. doi: 10.1093/nar/gkag652 (PMC13309788; doi:10.1093/nar/gkag652)
Supplement: gkag652_Supplemental_File [file gkag652_supplemental_file.pdf]

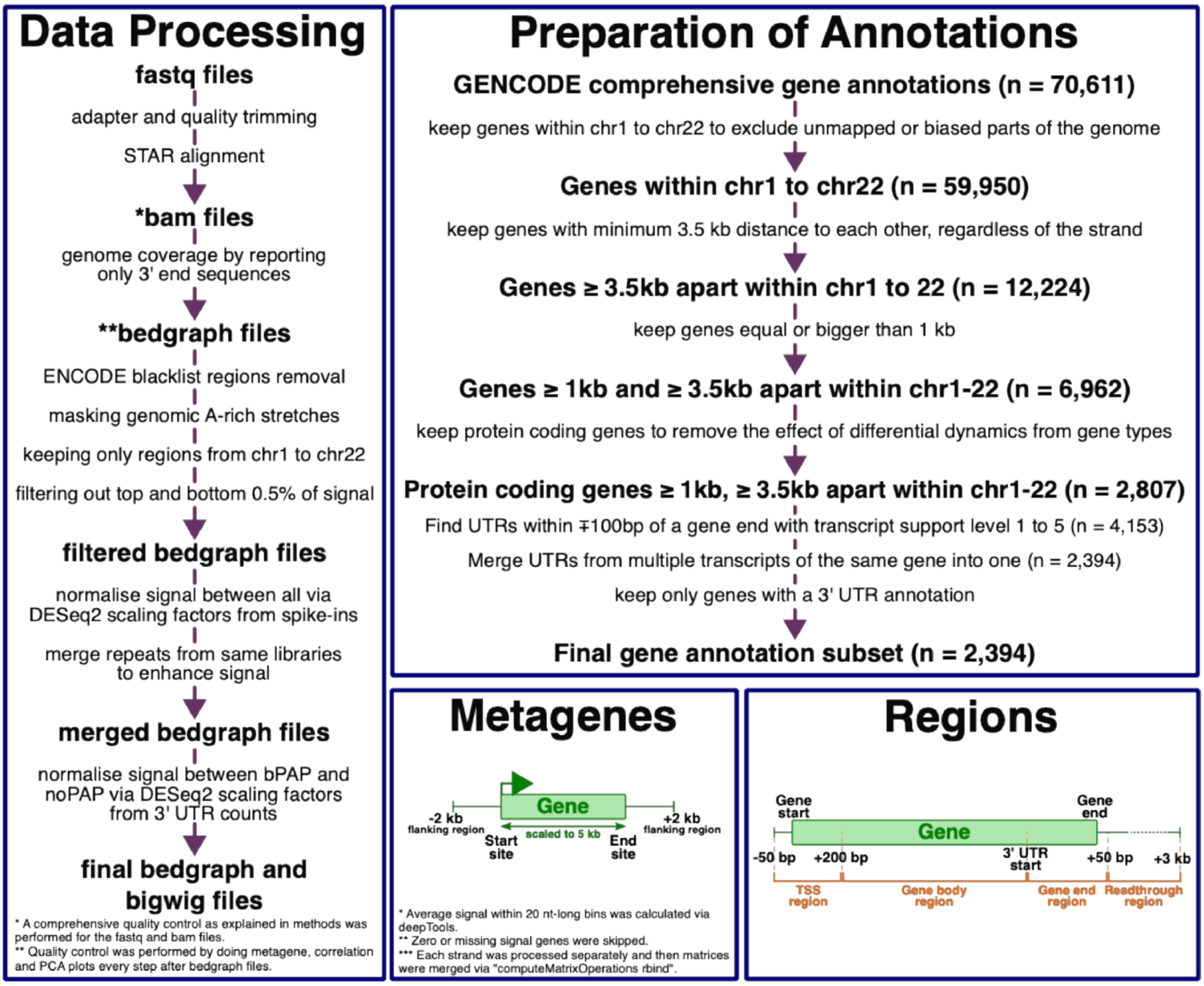

Supplementary Figure 1. Establishment of TT-Seq and SNU-Seq in HEK293 and Hep3B cells. Schematic of data processing, annotation preparation, metagene and region definitions.

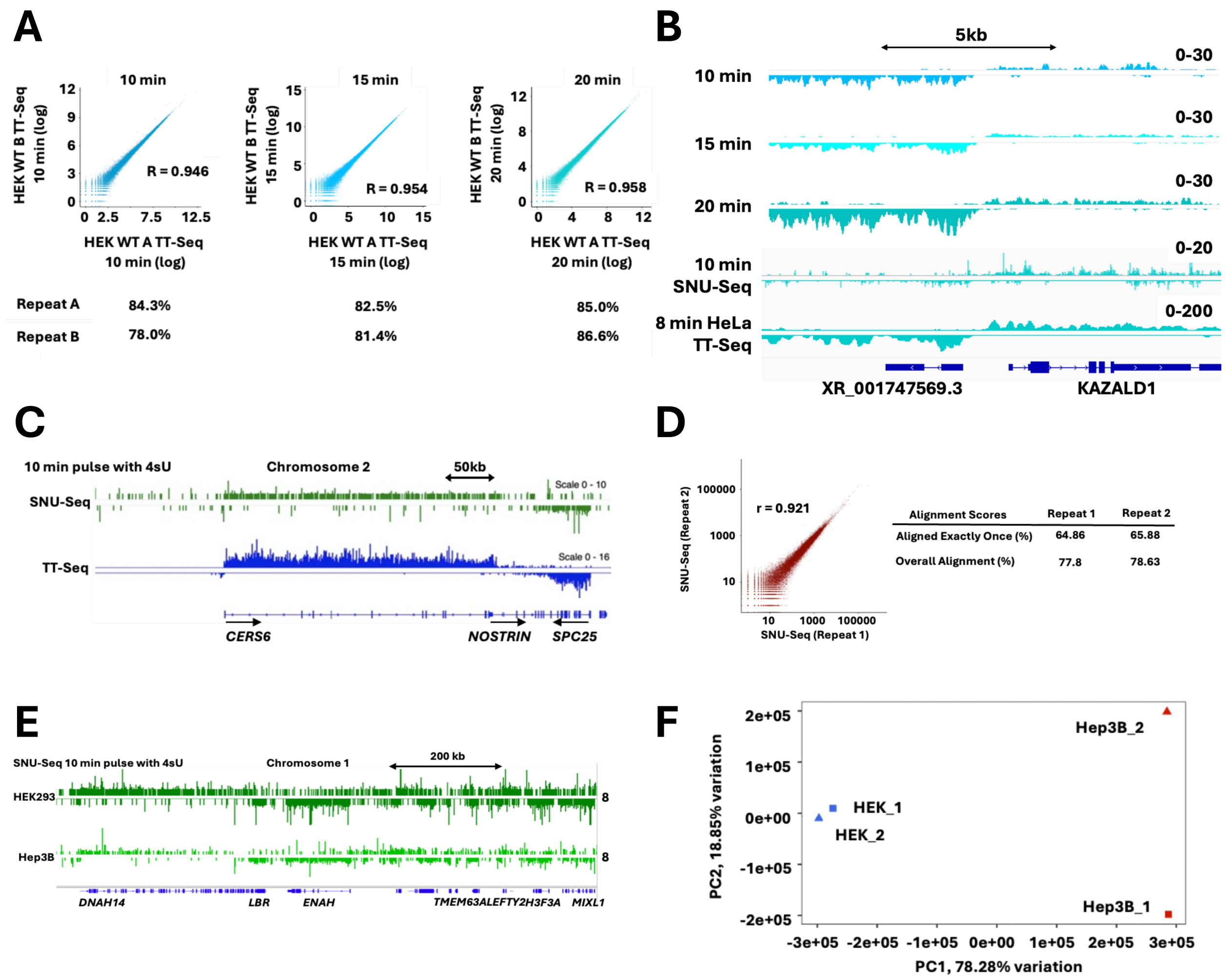

Supplementary Figure 2. Establishment of TT-Seq and SNU-Seq in HEK293 and Hep3B cells.

B Scatter plot of log-transformed read counts and alignment percentages for two repeats of 10, 15 and 20 min pulse-labelled TT-Seq experiment in HEK293 cells. The Pearson correlation coefficient (r) is shown. **C** IGV genome browser screenshot of TT-Seq and SNU-Seq output at the *KAZALD1* locus from HEK293 (10, 15, 20 min pulse-labelled TT-Seq, and 10 min pulse-labelled SNU-Seq) and HeLa cells (8 min pulse-labelled TT-Seq). **D** IGV genome browser screenshot of SNU-Seq and TT-Seq output in HEK293 cells (this study) on a region of chromosome 2. **E** Scatter plot and alignment score of SNU-Seq repeats read counts (log scale) in HEK293 cells. The Pearson

correlation coefficient is shown (r = 0.921). **F** IGV screenshot displaying a comparison of SNU-Seq in HEK293 and Hep3B cells on a region of chromosome 1. **G** Principal component analysis of SNU-Seq in HEK293 and Hep3B cells with two repeats each. The first (x-axis) and second (y-axis) principal components (PCs) are plotted against each other in a biplot. (Relates to **Figure 1**).

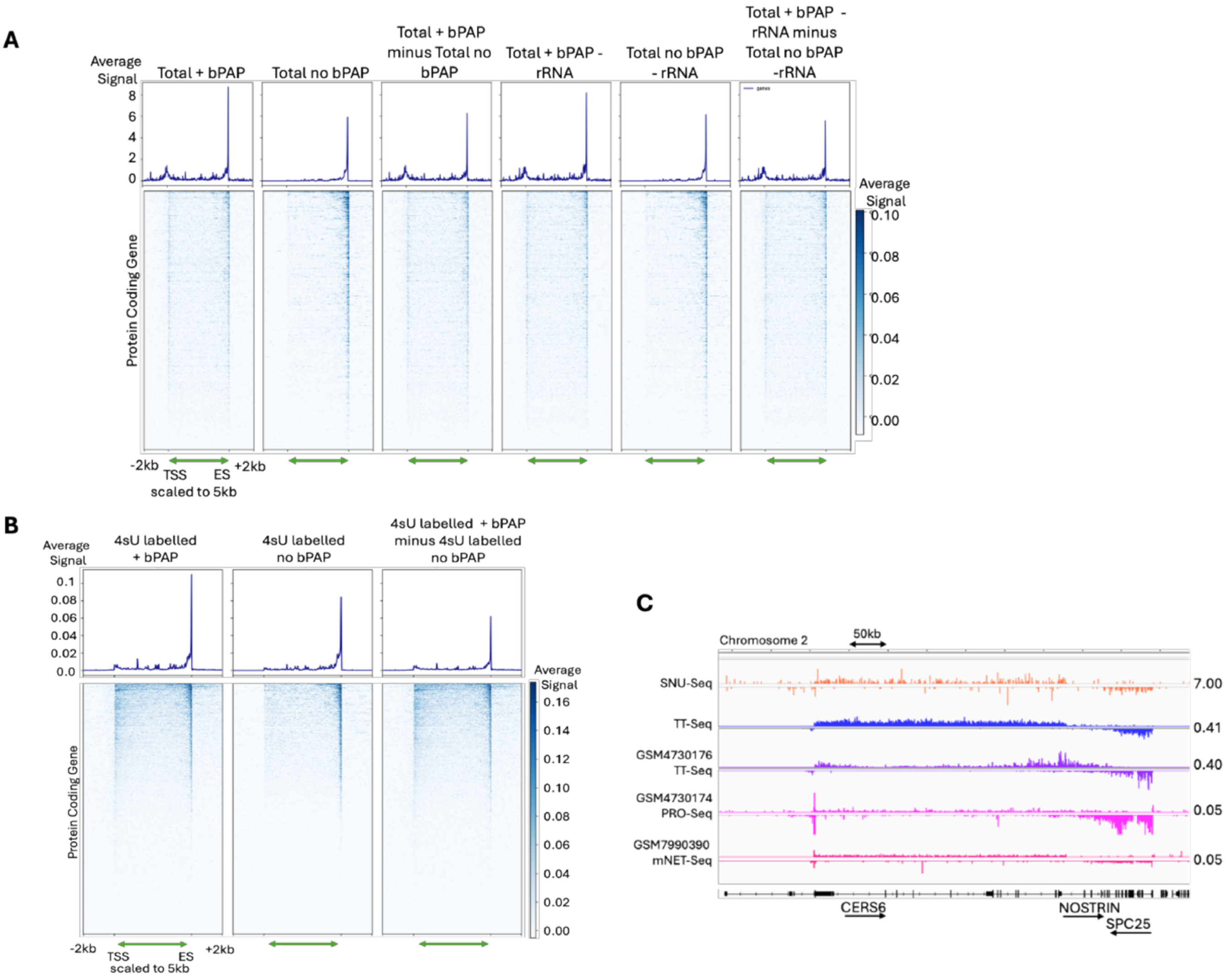

Supplementary Figure 3. **Single-nucleotide resolution 4sU-Seq (SNU-Seq) reports nascent transcription at high resolution**. **A,B** Metagene profiles and heatmaps for the transcription start site (TSS) and annotated end site (ES) of 2,394 separated GENCODE protein coding genes after blacklist, genomic A tracks and outlier filtering, scaled to 5 kb (green double-headed arrow), and the flanking 2 kb up and downstream shown as average normalised reads for (**A**) total RNA, treated with or without bacterial poly(A) polymerase (bPAP) and before (not denoted) or after (-rRNA) depletion of rRNA, and for (**B**) non-rRNA-depleted 4sU-labelled RNA treated with or without bPAP.

(**C**) Comparison of SNU-Seq (10 min 4sU pulse labelling n = 3), TT-Seq (10 min 4sU pulse labelling n = 2), TT-Seq, PRO-Seq and mNET-Seq in HEK293 cells around the *CERS6* locus. Sources of data, this study or as indicated. Note the PPP spikes at *CERS6* pre-mRNA and PDAT in SNU-Seq, PRO-Seq and mNET-Seq but not in TT-Seq data (Relates to **Figure 1**).

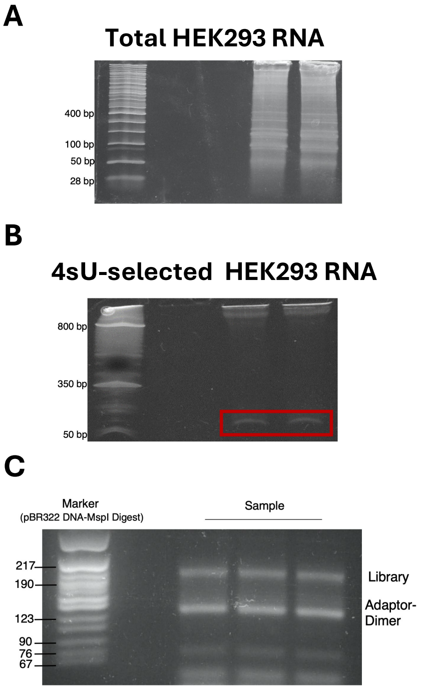

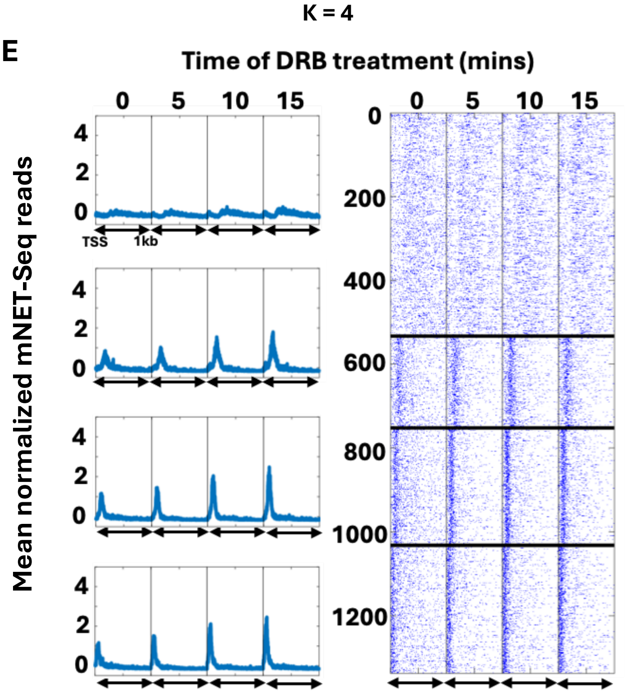

D

Sequencing Stats	Number	Percentage
Number of Total Reads	333,002,721	
Reads that align 0 times	44,549,628	13.3 %
Aligned Reads	288,453,093	86.7 %

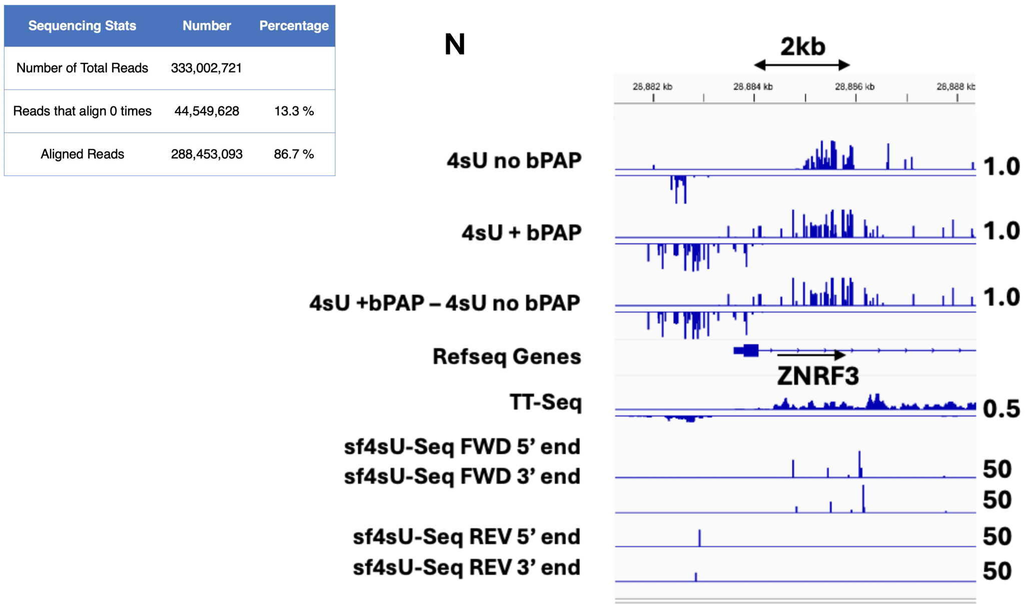

Supplementary Figure 4. Size-fractionated 4sU-Seq (sf4sU-Seq) 3′-end captures the promoter proximal pause: Data Analysis. **A-B** 3.5% TBE-urea gels run with (**A**) total and (**B**) isolated 4sU-labelled RNA from HEK293 cells. **C** DNA agarose gel of the prepared sf4sU-Seq library, showing products after adaptor ligation and PCR amplification. The top bands represent the desired library product, whereas the second-highest bands are a result of adaptor dimerisation. **D** Sequencing depth and mapping efficiency (percentage) of sf4sU-Seq reads. **E** Heatmaps and metagenes over the first 1000 nucleotide of genes, showing four k-means clusters (*k* = 4) of normalised HeLa mNET-Seq reads before and after treatment with the CDK9 inhibitor 5,6-dichloro-1-beta-D-ribofuranosylbenzimidazole (DRB) for the time indicated (0, 5, 10, 15 min), resulting in accumulation of RNA polymerase at the 5′ end of genes (1). The horizontal lines on the heatmaps indicate the transitions between the clusters. The numbers indicate the number of loci that fall into the four clusters. **N** IGV snapshot of reads around *ZNRF3*, illustrating early termination and polyadenylation of promoter proximal pre-mRNA and divergent PDAT transcripts. TT-Seq, SNU-Seq and sf4sU-Seq outputs for the 5′ and 3′ ends on each strand. Read scales for each library are shown for comparison.

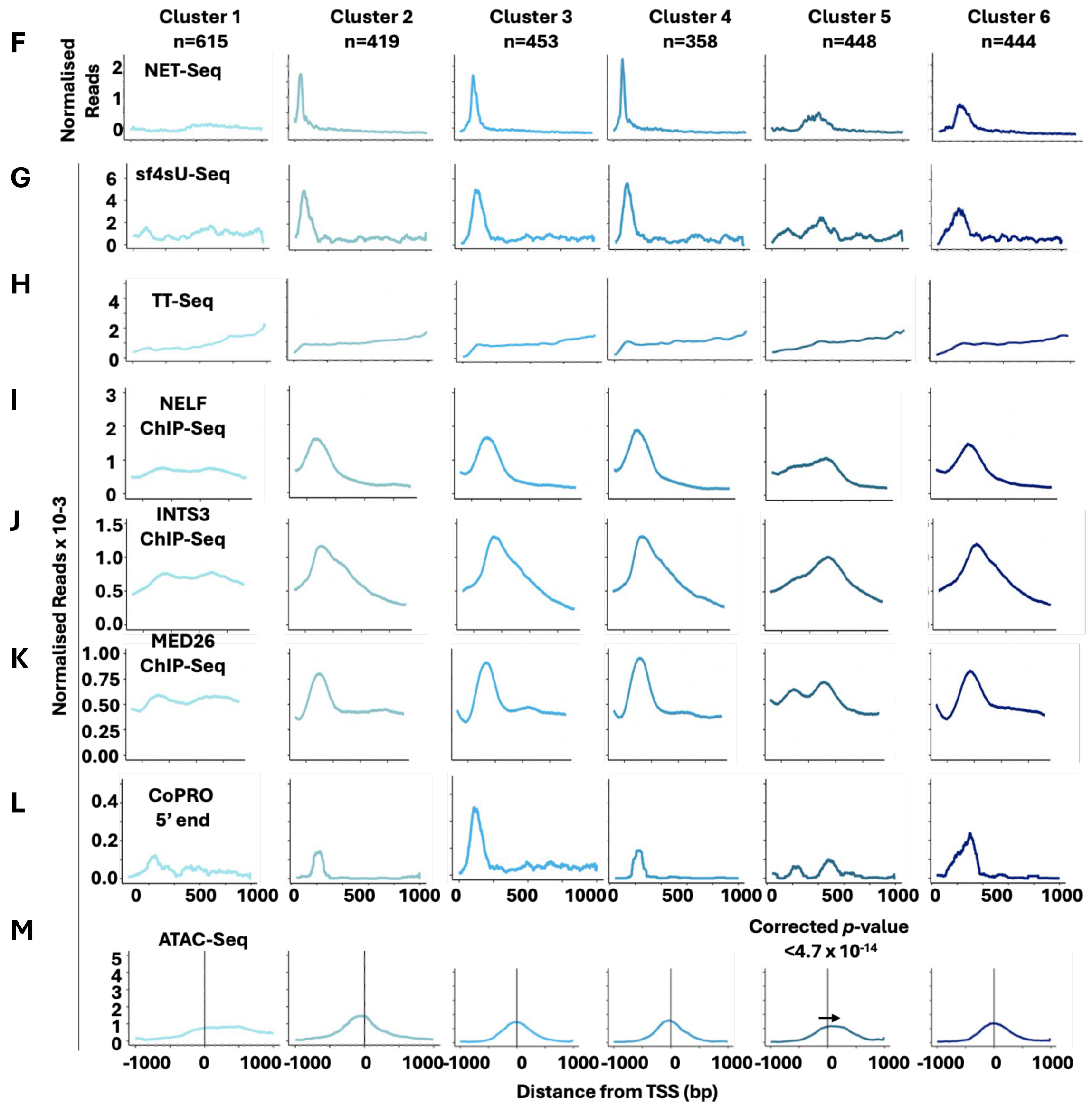

Supplementary Figure 4. Size-fractionated 4sU-Seq (sf4sU-Seq) 3′-end captures the promoter proximal pause: Data Analysis. **F-M** The *k*-means clusters 1 to 6 based on the shape of HeLa mNET-Seq data over the first 1000 nt of genes (scaled). Metagenes of the NELF, INTS3, MED26 ChIP-Seq (HeLa cells), TT-Seq (HEK293 cells) and CoPRO (K562 cells) (2) data were also plotted for these clusters. Position of ATAC-Seq peaks ± 1kb (HEK293 cells) (3) were also demonstrated for these clusters. Only the peak in Cluster 5 had a minor but statistically significant shift from the TSS (adjusted p < 4.7 x 10^{-14}). (Relates to **Figures 1 and 2)**.

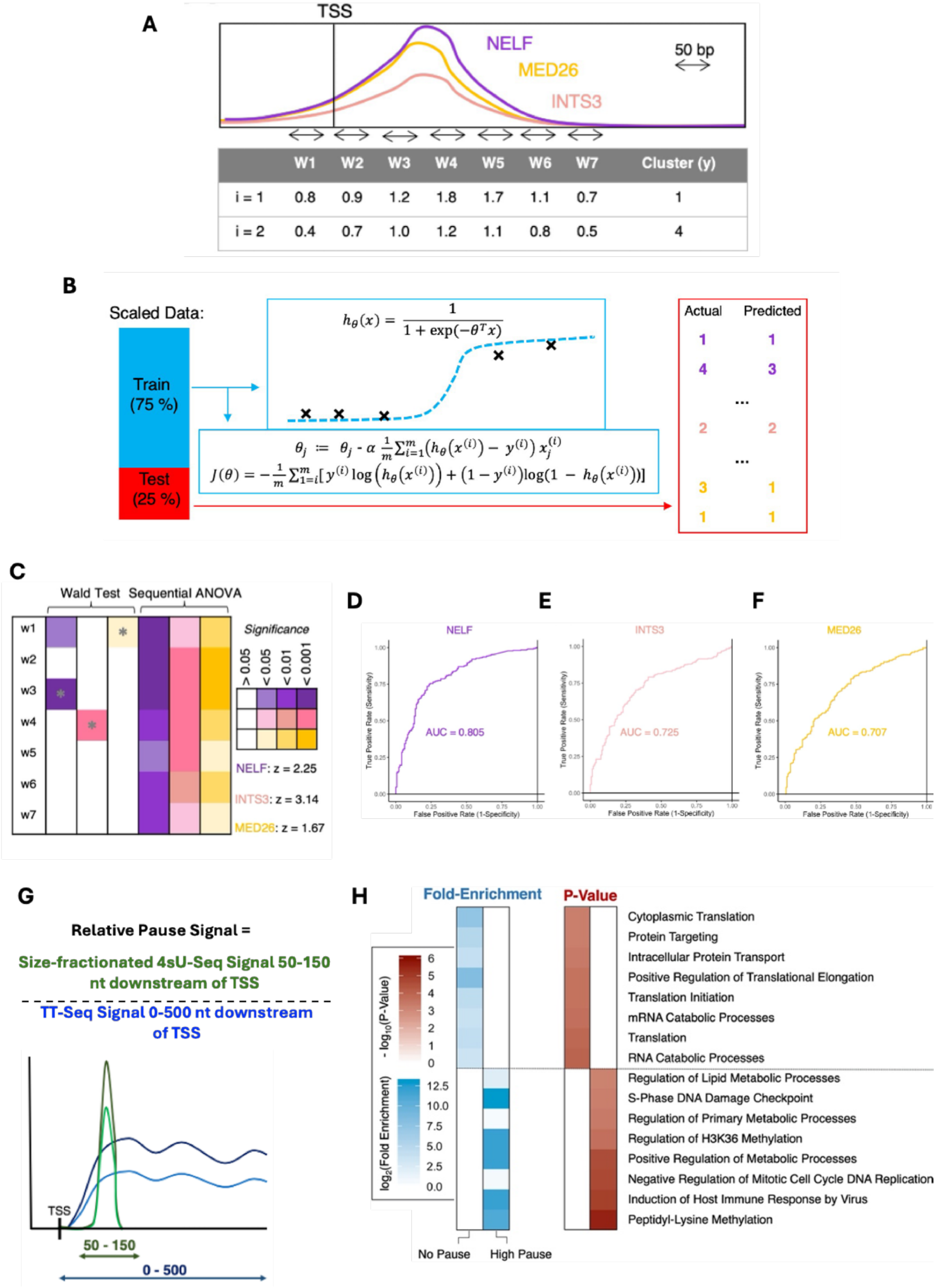

Supplementary Figure 5: Size-fractionated 4sU-Seq (sf4sU-Seq) 3′ end captures the promoter proximal pause: Modelling. A Preparation of NELF, integrator, and

mediator ChIP-Seq data to be able to use in training and testing a machine learning model for predicting the four mNET-Seq-based clusters found in a previous k-means analysis. Sums of normalised signal for 50 bp-long windows, starting from one window upstream (W1) and six windows downstream of the transcription start site (TSS) of genes (W2 to W7, covering promoter proximal pause site), were calculated as the input. The data were scaled to ensure equal contributions from genes of different lengths. **B** The data, prepared as described in **A,** were split into a training (75%) and a test (25%) dataset. The sigmoid hypothesis function of the logistic regression model (upper blue box) and the related gradient descent and cost function equations (lower blue box) are shown. m denotes the total number of training examples (total number of genes, i.e., 1845). J denotes the cost function, i denotes the training example (i.e., the gene), and j denotes the feature (i.e., the window). The vector containing the 7 parameters is termed θ. $x^{(i)}$ is a vector containing the summed ChIP-Seq signal for the i-th gene in the 7 windows. $y^{(i)}$ denotes the value to be predicted, i.e., the cluster. h is the hypothesis function. Following training, the model was evaluated on the held-out test dataset to assess its performance by comparing actual and predicted clusters (red box on right). **C** *p*-values associated with the probability of coefficients being significantly different from zero are displayed for NELF (purple), INTS3 (pink), and MED26 (gold), as determined by the Wald test, which tests whether each window's contribution can be removed without significantly affecting model predictions. *p*-values in the left half of the table are determined by ANOVA, which sequentially compares the model containing all the previous variables to the one that additionally includes the variable in question.

z-values indicate the magnitude of this coefficient's influence on classifying the data points into clusters 2-4 when it is positive. A negative number indicates that higher levels in this window are more likely to classify the gene as belonging to cluster 1. The window to which the maximum z-values belong is indicated with a grey star. **D-F** True positive rate (sensitivity) vs false positive rate (1 – specificity) plot and the area under the curve (AUC) for the (**D**) NELF, (**E**) INTS3 and (**F**) MED26-trained logistic regression models, assessed with the held-out test dataset. **G** Calculation of the relative level of the promoter proximal pause for the analysis in **H**. **H** Fold-enrichment and p-values of gene ontology analysis by GORILLA are shown for genes that exhibit high levels of calibrated pausing (top decile) or no pausing (bottom decile). log_2-transformed fold-enrichment values and negative log_{10}-transformed p-values are shown. The top and bottom deciles each contained 934 genes (out of a total of 9,336). Genes with zero TT-Seq or zero SNU-Seq reads were ignored. (Relates to **Figure 2**).

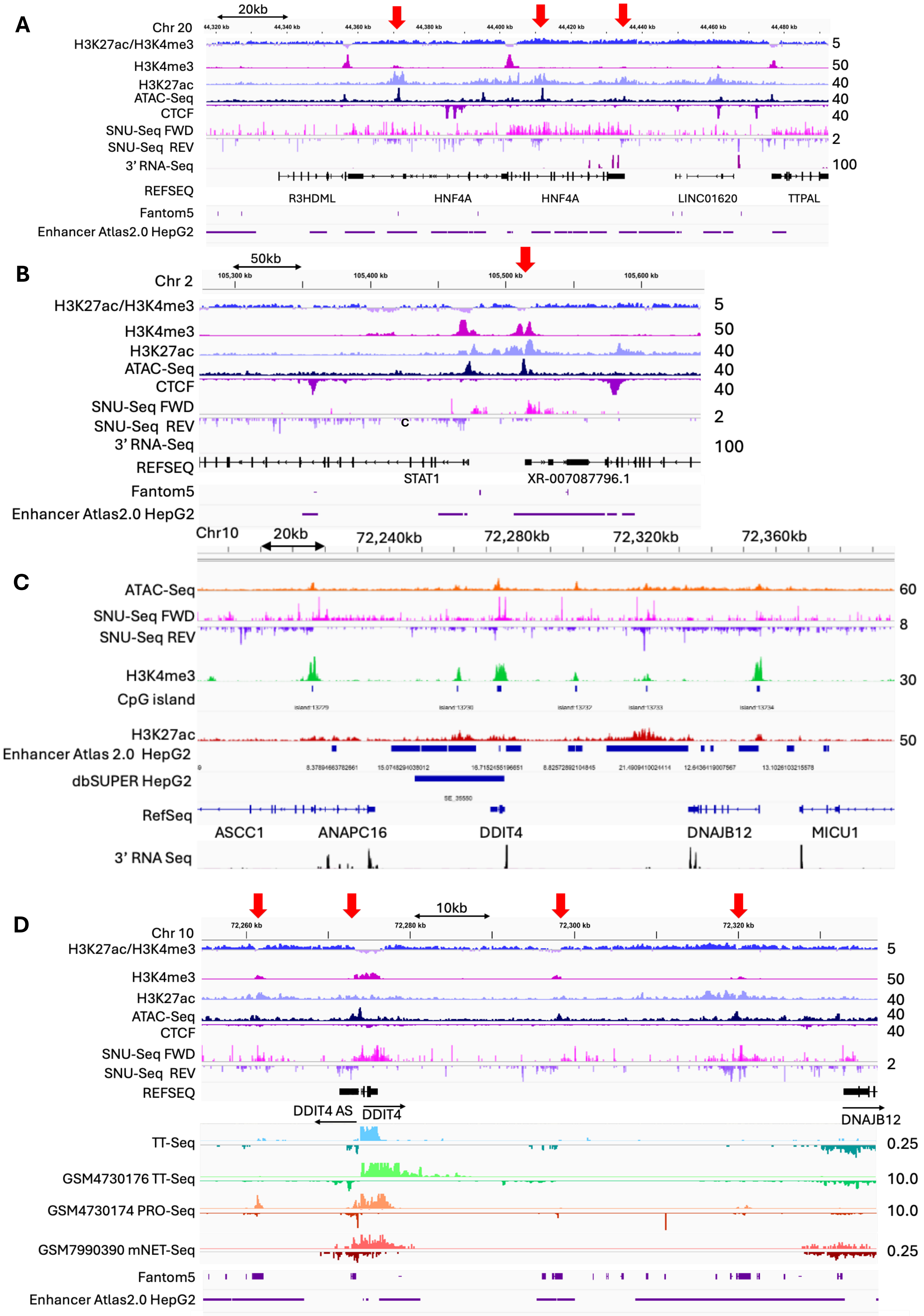
A
Chr 20
20kb
H3K27ac/H3K4me3
H3K4me3
H3K27ac
ATAC-Seq
CTCF
SNU-Seq FWD
SNU-Seq REV
3' RNA-Seq
REFSEQ
Fantom5
Enhancer Atlas2.0 HepG2
R3HDML
HNF4A
HNF4A
LINC01620
TTPAL
B
Chr 2
50kb
STAT1
XR-007087796.1
C
Chr10
20kb
72,240kb
72,280kb
72,320kb
72,360kb
ATAC-Seq
SNU-Seq FWD
SNU-Seq REV
H3K4me3
CpG island
H3K27ac
Enhancer Atlas 2.0 HepG2
dbSUPER HepG2
RefSeq
ASCC1
ANAPC16
DDIT4
DNAJB12
MICU1
3' RNA Seq
D
Chr 10
10kb
DDIT4 AS
DDIT4
DNAJB12
TT-Seq
GSM4730176 TT-Seq
GSM4730174 PRO-Seq
GSM7990390 mNET-Seq
Fantom5
Enhancer Atlas2.0 HepG2

Supplementary Figure 6. The chromatin environment and transcription in Hep3B cells. A-D IGV snapshots showing chromatin features and nascent transcription around loci expressed in hepatocytes with annotated enhancers (including FANTOM5 and Hep2G Enhancer Atlas annotations) indicated by red arrows for (**A**) *HNF4A*, (**B**) *STAT1* and (**C,D**) *DDIT4*, also including a comparison of SNU-Seq (10 min 4sU pulse labelling n = 3), TT-Seq (10 min 4sU pulse labelling n = 2), TT-Seq, PRO-Seq and mNET-Seq in HEK293 cells from sources indicated for *DDIT4*. Scales are indicated with the numbers on the side. (Relates to **Figure 6**).

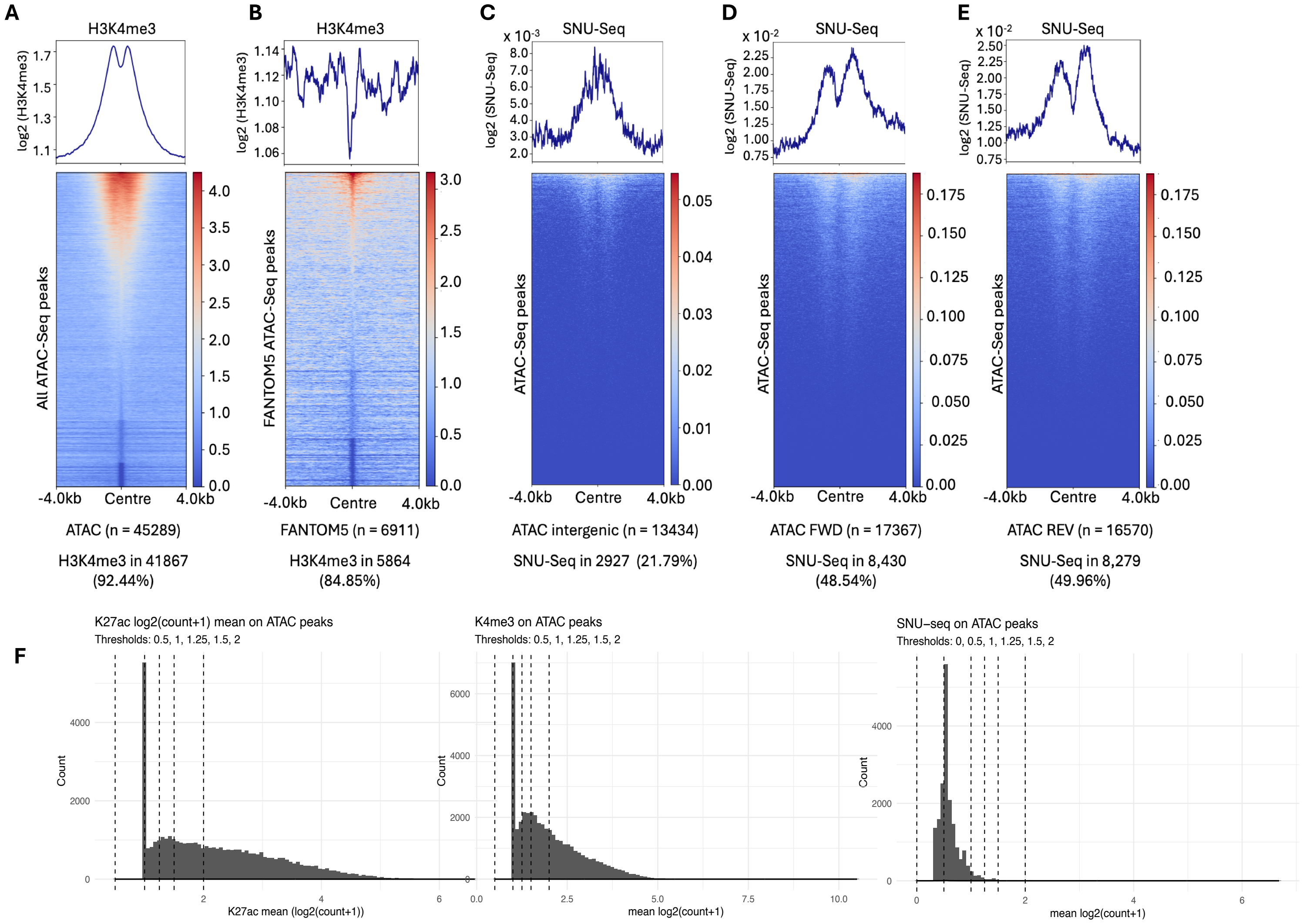

Supplementary Figure 7. The chromatin environment and transcription in Hep3B cells. A-E Metagenes and heatmaps showing (**A,B**) H3K4me3 or (**C,D**) SNU-Seq distribution around all (**A**) ATAC-Seq peaks **(**n = 45,289)**,** (**B**) FANTOM5 annotations (n = 6,911), (**C**) intergenic ATAC-Seq peaks (n = 13,434) or (**D,E**) ATAC-Seq peaks associated with promoters in a stranded way. **F** Setting thresholds for levels of H3K27ac, H3K4me3 or SNU-Seq for inclusion in **Figure 6G**. (Relates to **Figure 6).**

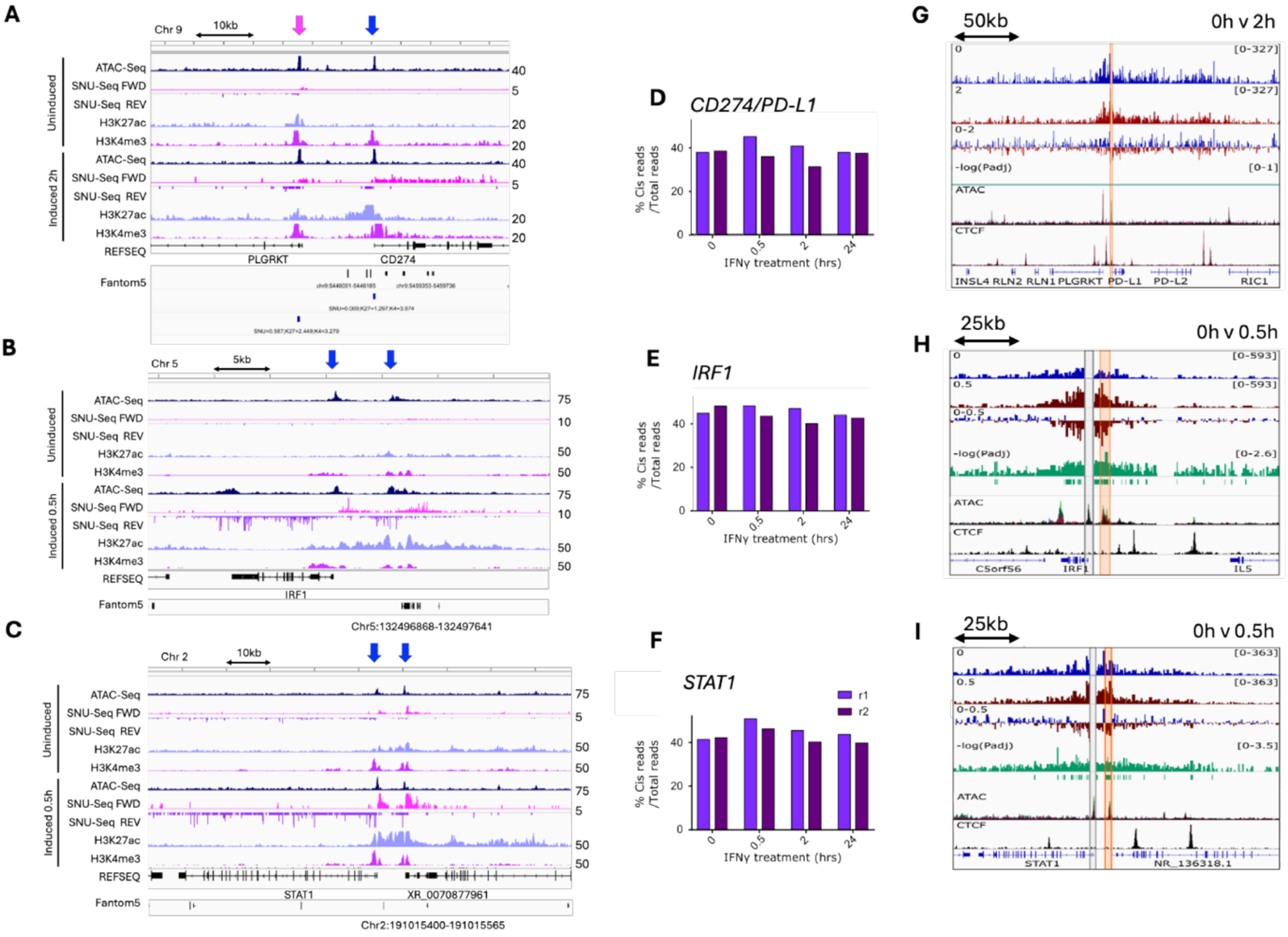

Supplementary Figure 8. Long-range chromatin interactions at genes with IFNγ-inducible transcription in Hep3B cells. **A-C** IGV snapshots showing chromatin features and nascent transcription around *CD274*, *IRF1* and *STAT1* before or after induction with IFNγ. Promoters and enhancers (blue arrows) or putative enhancers (pink arrow) are indicated. The read scale are indicated. **D-F** Bar charts showing the percentage of *cis* reads over the total reads for each Capture-C sample and repeat at 0, 0.5, 2 and 24h of IFNγ treatment for the genes indicated. Similar ratios indicate similar library qualities. **G-I** Significant differential Capture-C interactions between the

STAT1 (0 v 0.5h induction), *IRF1* (0 v 0.5h induction) and *CD274* (0 v 2h induction) promoters (position of the probe is shown by grey bar) and putative enhancers (marked with orange bar for *STAT1* and *IRF1*). Reads are counted per NlaIII-digested fragment. The tracks top to bottom show *cis*-normalised mean data for uninduced (0h; blue), then induced (0.5 or 2h; brown), then the differential signal (any signal below the line is enriched in induced cells), then the significance of the difference (-log(Padj)) with green bars. Normalised ATAC-Seq and CTCF (ChIP-Seq) tracks are shown. For *STAT1* and *IRF1*, the probe positions are highlighted in grey, and significant interactions with the known enhancer elements are highlighted in orange. For *CD274,* the probe position is indicated by the orange box. No significant changes are observed, as constitutive interactions occur with the *PLGRKT* putative Epromoter. (Relates to **Figure 9**).

Supplementary Materials and Methods

Cell Culture

HEK293 and Hep3B cells were cultured in DMEM, supplemented with 10% (v/v) FBS and 1% (v/v) Penicillin-Streptomycin (Sigma cat #P0781). The incubator was set to 37°C with 5% CO_2. HEK293 and Hep3B cells were grown in three 15-cm dishes up to ≈ 80% confluency (≈ 6 x 10^7 cells). Cells were counted on a Nexcelom Biosciences Auto 2000 Cell Counter or a BioRad TC10 Automated Cell Counter. Hep3B cells were passaged 48 hrs before harvesting at 80% confluency. After passage, they were left for 24 hrs to adapt, then left untreated or treated with 10 ng/ml of IFNγ for the time stated.

Preparation of spike-ins for SNU-seq and TT-seq

For all SNU-Seq in HEP3B cells, all TT-seq in HEK293 cells, and some of the SNU-seq in HEK293 cells (GSM5452294), three 1000-bp-long DNA fragments covering *Saccharomyces cerevisiae* genes *BAT2*, *HXT1* and *GAL1* were amplified using forward primers containing T7 promoter sequence. The thio-labelled spike-in RNAs were prepared by *in vitro* transcription (IVT) via the MEGAscript T7 Transcription kit using a 1:5 ratio of thio-UTP to UTP.

For the rest of the SNU-Seq and control samples from the HEK293 cells, the thio-labelled *Renilla* luciferase RNA was *in vitro* transcribed via HighYield T7 mRNA Synthesis Kit (Jena Bioscience RNT-101) using a 1:5 ratio of thio-UTP to UTP (Jena Bioscience NU-1156L) on the BspQI (NEB R0712)-linearised and phenol-chloroform-ethanol purified DNA template, including T7 promoter, treated with DNase I (NEB

M0303) and cleaned up using Monarch Spin RNA Cleanup Kit (NEB T2040L). Concentrations were determined using NanoDrop 2000 Spectrophotometer (Thermo Scientific) and its integrity confirmed by agarose gel electrophoresis.

TT-Seq

TT-Seq was performed as described in the original publication (4) with modifications. 60 million cells were incubated with 0.5 mM 4sU for 10 min (HEK293) or 5 min (HeLa) at 37°C. Cells were washed rapidly before being lysed in 0.7 mL QIAzol per 10 million cells, scraped into 2 mL tubes, homogenised by vortexing for 10 s after adding 2.4 ng of thio-labelled spike-in RNAs per million cells. Total RNA was purified using a 1:5 chloroform:QIAzol and isopropanol precipitation. Concentration was determined using a NanoDrop. 300 µg total RNA in 400 µL water was sonicated in a Bioruptor at the high setting for 1 cycle of 30s on / 30s off. Biotinylation of the thio-labelled RNA using EZ-link HPDP Biotin in dimethylformamide (400 µg) was done at room temperature for 2 hours with shaking/rotation in the dark. To purify the RNA, phase-Lock tubes were used with an equal volume of chloroform, then equal volume of isopropanol and precipitated with 1/10 volume of sodium chloride. µMACS streptavidin kit was then used to purify biotinylated-4sU-labelled RNA. 100 µL µMACS streptavidin beads were used per 200 µL biotinylated RNA, incubating at 4°C for 15 min with shaking/rotation, after columns equilibration. The RNA on the column was washed at three times at 65℃ and then at room temperature with 100 mM Tris pH 7.5, 10 mM EDTA pH 8.0, 1 mM NaCl, 0.1% Tween-20. The labelled RNA was eluted with 100 µL of 100 mM dithiothreitol (DTT), then, after waiting 5 min, eluted with another

100 µL of DTT, and RNA immediately purified using the miRNeasy kit. DNase-I treatment was performed on the column according to the manufacturer's instructions. Quality and quantity of the thio-labelled RNA were determined using Agilent's Bioanalyzer RNA Pico chip. Sequencing libraries were prepared using the NEBNext rRNA Depletion kit for Human Cells, followed by the NEBNext II Ultra Directional RNA Library Prep kit, according to both kit instructions. Library quality was assessed using Agilent's Bioanalyzer DNA-High Sensitivity chip. Pooled libraries were paired-end sequenced with 150 cycles on the Illumina NextSeq 500 platform using the High-Output Illumina NextSeq 500 kit.

TT-Seq Sequencing Analysis

Paired-end sequencing reads were quality-checked with FastQC, then trimmed using Trim-Galore for a Q score below 20 and aligned to the human genome (hg38) with HISAT2 (5) using the "–no-mixed" and the "-no-discordant" flags. Aligned files in the sam format were then filtered by using samtools (6) with the flags "-q 40, -f 99, -F3852, -bS". Calibration of samples (where necessary) was achieved by calculating DESeq2 scaling factors from spike-in counts across samples based on RNA counts tables generated by featureCounts (7). Bedgraph and bigwig files were generated from bam files using bedtools (8), and wigToBigWig (UCSC utility tools), respectively.

Single Nucleotide 4sU-Sequencing (SNU-Seq)

SNU-Seq was performed using the TT-Seq protocol but omitting the sonication step. Total RNA aliquots from the same samples for 3′ end RNA-Seq were first treated with DNase I and cleaned up using the Monarch Spin RNA Cleanup Kit (NEB T2040L). "No

4sU" libraries were prepared in the same way as SNU-Seq samples, except that water was added instead of 4sU. For the rRNA-depleted libraries, after streptavidin pulldown, rRNA depletion was performed using the NEBNext rRNA Depletion kit for Human Cells (NEB E6310S).

Before preparation of "bPAP" libraries, 150 ng of thio-labelled RNAs were polyadenylated using the NEB *E. coli* poly(A) polymerase (bPAP) for 45 min at 37°C before precipitation using an equal volume of isopropanol with 1/10 volume of sodium acetate and 0.5 µL GlycoBlue co-precipitant. The pellet was resuspended in 11-22 µL RNase-free water. Qualities and amounts of the labelled RNA were checked on the Thermo Fisher's Qubit RNA High Sensitivity and the Agilent's Bioanalyzer RNA Pico Chip or the Agilent's TapeStation RNA High Sensitivity ScreenTape. Total RNA was checked on the Thermo Fisher's Qubit RNA Broad Range and Agilent's TapeStation RNA ScreenTape.

Library preparation was performed by Lexogen GmbH (Vienna, Austria). Briefly, libraries were prepared by using the maximum RNA input amount (5 µL) following the Quant-Seq Lexogen 3' mRNA kit (Ion Torrent or FWD for Illumina samples) instructions with 13 PCR cycles. Library qualities were checked using Agilent's Bioanalyzer DNA High Sensitivity chip or Agilent's Fragment Analyzer High Sensitivity Genomic DNA chip. Chips were prepared using the Ion Chef and sequenced on an Ion Proton Sequencing platform or by Lexogen GmbH's (Vienna, Austria) services on an Illumina NextSeq2000 platform. The samples were single end sequenced with 100 nt read length (SR100). Different read depths were tested, such as ~5 M for one batch and

~50 M for another, as a comparison. Notably, both read depths yield similar results: 50 M had more duplicates but also captured more non-coding transcripts than 5 M, suggesting an optimal read depth of around 20-30 M, validated in other replicates.

SNU-Seq - Sequencing Analysis

After quality control with FastQC, FastQ files were trimmed with Trimmomatic (9) or bbduk.sh (BBTools) to remove reads with a quality score below 20 in a sliding window of 5 bp and the poly(A) reads. Sequences were aligned with HISAT2 with the same settings as for TT-Seq, or with STAR using "--outSAMtype BAM SortedByCoordinate --outSAMunmapped Within --outSAMattributes All --outFilterMultimapNmax 10 --winAnchorMultimapNmax 50 --alignSJoverhangMin 8 --alignSJDBoverhangMin 1 --outFilterMismatchNmax 10 --outFilterMismatchNoverReadLmax 1 --outMultimapperOrder Random --alignEndsType EndToEnd --alignIntronMin 11 --alignIntronMax 0 --alignMatesGapMax 0". Sorted BAM files were generated using samtools. For alignment, hg38 (GrCh38.p14) was used together with GENCODE (v46) annotations. Genomic A stretches were masked using the criteria: all regions with ≥ 4 A within 6 nt but no C or T residues downstream or ≥ 12 A within 18 nt downstream of a given position, and all regions with ≥ 15 A within 18 nt upstream of a given position (10), resulted in masking ≈ 2.8% of the hg38 genome, including scaffolds, alternate loci and assembly patches. These masked regions, together with the blacklist regions published by ENCODE (11) were then removed from each BAM file via bedtools intersect -v function. Outliers were removed by determining the top and bottom 0.5% of the signal. Calibration of samples (where necessary) was achieved by calculating

scaling factors from spike-in counts between samples via DESeq2, based on the RNA counts table generated by featureCounts. Bedgraph and bigwig files were generated from bam files using bedtools, and wigToBigWig, respectively. 3′ end single nucleotide coverage was achieved by using the bedtools genomecov function's "-3" option. A further normalisation for no bPAP bedgraph and bigwig files was done by using 3′ UTR counts obtained using bedtools map function's "-o sum" option.

Size fractionated 4sU-Seq (sf4sU-Seq)

The thio-labelled RNA was generated as described for SNU-Seq and run on a 3.5% TBE-Urea gel (8 M urea) after adding 2X loading dye and heated to 80 °C for 2 minutes. The gel was kept on ice and pre-run for 10 min at 80 V, using ice-cold ultra-pure TBE (1X) as the running buffer washing the wells with 1X TBE before loading the samples and running at 80 V for 90 minutes. The gel was transferred to a square petri dish and incubated in 1X ultra-pure TBE with SYBR Gold (1/10,000 v/v) for 5 minutes. The gel was rinsed twice in 1X ultra-pure TBE before imaging. The small thio-labelled RNA gel region was cut using a razor blade and incubated at -80°C for 10 minutes, then extracted using dialysis: the gel slice, along with 0.8 mL 10 mM Tris-HCl (pH 7), was placed into SnakeSkin dialysis membrane sealed by two Eppendorf caps. The dialysis was run for 35 min at 80 V. The RNA was precipitated with isopropanol and eluted in 11 µL RNase-free water. Quality and size distribution of the size-selected RNA were checked on Agilent's Bioanalyzer RNA Pico chip.

The size fractionated thio-labelled RNA was decapped with 5′ Pyrophosphohydrolase (NEB) in ThermoPol® reaction buffer following the manufacturer's instructions. The

reaction was stopped with 1 µL 500 mM EDTA and heat-inactivated by incubation at 65°C for 5 min. RNA was precipitated with isopropanol and resuspended in 6 µL RNase-free water. Libraries were generated using the NEBNext Small RNA Library kit for Illumina following the kit instructions. Library quality was assessed with Agilent's Bioanalyzer DNA High Sensitivity chip. Sequencing was performed on an Illumina NextSeq 500 platform. After sequencing, fastq files were quality-trimmed with Trim-Galore and aligned with bowtie2 (12). Sorted BAM files were generated from aligned SAM files using samtools with a filtering step added to only retain reads that have a mapping quality score above 30 (-q 30). 3′ end single-basepair resolution was extracted using the bedtools genomecov's -3 option when generating bedgraph files. To generate TSS annotations from the sf4sU-Seq samples, 5′ end coverage was generated using the bedtools genomecov's -5 option (instead of -3) when generating bedgraphs from bam files in bedtools. To generate 5′ end reads that only occur in nucleosome-depleted regions, ATAC-Seq data in HEK293 cells (3) (E-MTAB-6195) were used. The ATAC-Seq fastq files were trimmed with Trim-Galore, aligned with bowtie2, and converted to bam and bed files using samtools and bedtools, respectively. Subsequently, MACS2 was used to call peaks (no model assumed). The intersect option in bedtools was then used to retain only those 5′ ends from sf4sU-Seq signals that are located within ATAC-Seq peaks. The resulting bedgraph files were then used as an input to identify TSS cluster centres using Paraclu (13), which identifies clusters in a sliding window, and a cluster value threshold of 30 was applied. Cluster-centres were calculated using the mean position of the cluster, and TSS candidates were verified in

MATLAB as follows: only those TSS candidates were assigned as true active TSSs where the summed TT-seq signal (using the 10-min labelled HEK293 TT-Seq sample) in the 1000 bp downstream of the candidate TSS was at least 5 times greater than in the 1000 bp upstream of the candidate TSS. TSScall, a Python script (14), was then used to identify annotated and unannotated (novel) TSSs using the comprehensive GENCODE (v29) annotation file, yielding 2,955 previously annotated TSSs and 1,428 novel TSSs. The read threshold for this was set to 5, which corresponded to an FDR of 0.001.

Annotation Preparation for Metagene Analysis and Mathematical Modelling

For an unbiased metagene representation, the averaged regions are separated from other possible signals. GENCODE (v46) comprehensive gene annotations (n = 70,611) were filtered to retain genes within chr1 to chr22 to exclude the biased/unmapped regions of the reference genome (n = 59,950). Then, only genes with a minimum distance of 3.5 kb between them, regardless of the strand, were kept (n = 12,224). From this subset, only the protein-coding genes ≥ 1 kb were retained (n = 2,807). Finally, UTRs within ±100 bp of a gene end with a transcript support level 1 to 5 were detected, and they were merged if multiple PAS/UTRs were present for a gene. Only the genes with a 3′ UTR annotation were kept (n = 2,394). This is the GENCODE annotation subset that is used for metagene analysis and mathematical modelling.

Mathematical Modelling and Determination of Transcription Constants

Synthesis rates (SR) and decay rates (DR) were determined based on a previously published modelling approach (4,15): $DR = -(1/time)^* \log(1-RNA_{4sU}/RNA_{total})$, and $SR = RNA_{total}{}^*DR$, where time for labelling is 10 min, RNA_{4sU} is the normalised SNU-Seq

gene body (TSS +200 nt to the 3′ UTR start site) counts with bPAP treatment, exploiting the total RNA signal and the thio-labelled RNA signal from the same samples or HEK293 total or 3′ end RNA-Seq counts from (16). The pausing index was calculated as described (17), with a slight modification to the distances, defined as the ratio of normalised TSS (from -50 nt to +200 nt) counts to gene body counts.

Metagene and Data Analysis

deepTools was used for metagene calculation and visualisation. The “scale-regions” mode was used to compute metagene matrices with a 5 kb gene body length and 2 kb flanking regions, a 20 nt bin size achieved by averaging. Missing or zero-valued data were skipped. Each strand was calculated separately with strand-specific annotations, then merged with “computeMatrixOperations rbind” before visualisation. “computeMatrixOperations subset” was used to subset the matrix for the libraries of interest. *p*-values were determined using the non-parametric Wilcoxon rank sum test, and the Bonferroni correction for multiple testing was applied when required. For correlations, Pearson (*r*) was used for the correlation between repeats based on the counts, while Kendall (τ) was used for the correlation between synthesis rates and counts, and Spearman (ρ) was used in all other cases. Principal Component Analysis (PCA) and data visualisation were performed using deepTools (multiBigwigSummary followed by plotPCA) or in R using the PCAtools and ggplot2 with ggpubr packages, respectively. To compare the splicing levels in SNU-Seq and total RNA libraries, SPLICE-q (18) was used to calculate splicing efficiency (SE) from the BAM files. The SE score refers to the number of splicing events based on the ratio of spliced to unspliced reads

found around the splice junctions. The SE score (between 0 and 1) with values closer to 1 indicating a greater number of splicing events. The mean SE per library was computed to improve comparisons between libraries. To investigate the effect of the background signal (no 4sU) and the host polyadenylation signal during the pulse labelling window (no bPAP), a signal subtraction was performed for each nucleotide in the genome. For this exhaustive subtraction, deepTools bigwigCompare was utilised with "--operation subtract --binSize 1" parameters.

Nuclear and Cytoplasmic RNA Extraction

Extraction of RNA from nuclear and cytoplasmic subcellular fractions of HEK293 cells (n = 3) was as described (19). QuantSeq 3′ mRNA-Seq Library Prep Kit for Ion Torrent (Lexogen) was applied for nuclear (500 ng input) and cytoplasmic RNA (1,700 ng input) using 13 PCR cycles. Reads were aligned to the genome build using the Ion Torrent Server TMAP aligner with default alignment settings (-tmap mapall stage1 map4). Human poly(A) site (PAS) annotations were obtained from PolyA_DB3 (20). Each PAS was extended 20 nt 3′ and 200 nt 5′ from the site of cleavage, and those that overlapped on the same strand after extension were combined into a single PAS annotation. Mapped reads were narrowed to their 3′ most nucleotide and those which overlapped with the extended PAS annotations were counted. PASs associated with non-coding RNAs and genes not in the RefSeq (21) gene database were excluded. Genes with only one PAS were also excluded.

Chromatin analysis in Hep3B cells

ATAC-Seq was performed as previously described (22). 5 x 10^6 Hep3B cells were washed in cold PBS and resuspended in lysis buffer (10 mM Tris-HCl pH 7.4, 10 mM NaCl, 3 mM $MgCl_2$, 0.1% IGEPAL). The nuclei were pelleted and resuspended in 1X TD with 2.5 µL TDE1 (Nextera XT DNA library prep kit, Illumina). These were incubated for 30 min at 37°C. The tagmented DNA was purified using the MinElute PCR purification kit (Qiagen, 28004). Each sample was amplified using the Nextera XT DNA library prep kit and Nextera XT index kit (Illumina) with the following thermocycler programme: hold at 72°C (5 min), 98°C (30 s), 9 cycles of 98°C (10 s), 63°C (30 s), 72°C (30 s) with a final 72°C extension for 1 min. The libraries were purified by adding 1.8X volume of RT AxyPrep beads (AxyPrep Mag PCR cleanup kit) with a ratio of 0.6:1 beads, to remove fragments larger than 600 bp. Libraries were run on the Agilent's Bioanalyzer DNA High Sensitivity chip to assess quality. Paired-end sequencing was performed on a NextSeq 500 with the 75 cycles NextSeq 500/550 High Output v2 kit (Illumina). FastQCs were performed on each dataset to ensure the run was of good quality. The adapter sequences were trimmed and paired using Trimmomatic, removing reads with a quality score below 20 in a 5-bp sliding window and those shorter than 30 bp (9). Trimmed reads were aligned to the hg38 genome if un-spiked, or to a combined hg38-dm6 genome if spiked, using Bowtie2 (12). samtools were used to remove duplicates and filter out reads with a MAPQ quality score < 30, as well as mitochondrial reads (6). MACS2 was used to call peaks with a minimum FDR (q-value) of 0.01 (23). Following this, ENCODE blacklisted regions were removed (11). For tract visualisation, reads were

normalised by sequencing depth. Filtered BAM files were converted to bedgraphs using bedtools (8).

The ChIPmentation protocol used is largely based on the protocol published by Schmidl et al. (24). ≈ 10^6 cells were washed and collected in cold PBS. The cell pellet was resuspended in room temperature PBS and fixed for 5 min with 1% formaldehyde before quenching with 0.125 M Glycine. 5,000 fixed *Drosophila* Sg4 cells (0.05%) were added as a spike-in control. The cell pellet was obtained, washed in cold PBS and incubated on ice in cold swelling buffer (10 mM Tris-HCl pH 8, 10 mM NaCl, 0.2% NP-40, 1 mM AEBSF and 1X complete mini EDTA-free proteasome inhibitor, Roche) for 10 min. The subsequent nuclear pellet was resuspended in cold lysis buffer (10 mM Tris-HCl pH 8, 1% NP-40, 0.5% Na-deoxycholate, 0.1% SDS, 1 mM AEBSF and 1X complete mini EDTA-free proteasome inhibitor, Roche) and sonicated for 90 min, 30 s on and 30 s off, at 4°C (Bioruptor Plus, Diagenode). One tenth of the sample was taken as an input control. The remaining sample was incubated overnight, rotating at 4°C with the antibody. Scaling factors were calculated based on spike-in counts between samples or based on sequencing depth for CTCF ChIP, and used when converting the filtered BAM files to bedgraphs using bedtools (8). Bedgraphs were further converted to BigWig files using bedGraphToBigWig (UCSC utility tools). Differential analysis was performed between two datasets on non-normalised data using a custom R script that employed the DESeq2 package (25). To identify differential peaks rather than differential gene expression, peak files for each repeat/sample were merged into a

single file in which reads were counted using featureCounts and differentially assessed (7). A cut-off threshold of FDR ≤ 0.05 was used to define statistical significance.

Sequencing Data

Dataset (+ Citation)	Cell Line	Accession / Source
RNA-Seq Nuclear and Cytoplasmic	HEK293	This Study/GSE165251
SNU-Seq and Controls	HEK293	This Study/GSE179306
SNU-Seq	Hep3B	This Study/GSE172053
TT-Seq	HEK293	This Study/GSE179306
TT-Seq (4)	K562	GSE75792
TT-Seq	HeLa	This Study/GSE179306
TT-Seq (26)	HEK293	GSE156400
mNET-Seq (27)	HEK293	GSE251941
PRO-Seq (26)	HEK293	GSE156400
ATAC-Seq (3)	HEK293	E-MTAB-6195
RNA-Seq (16)	HEK293	E-GEOD-57027
3’RNA-Seq	Hep3B	This study/GSE172053
ATAC-Seq	Hep3B	This study/GSE172053
ChIPmentation	Hep3B	This study/GSE172053
sf4sU-Seq	HEK293	This study/GSE179306

Kits and Reagents

Kit / Reagent Name	Provider	Code
NEBNext rRNA Depletion Kit (Human/Mouse/Rat)	NEB	E6350S
Agencourt RNAClean XP	Beckman Coulter	A63987
NEBNext Multiplex Oligos (Set 1)	NEB	E7335L
NEBNext Ultra II Directional RNA Library Prep	NEB	E7765S
NEBNext Small RNA Library Prep Kit	NEB	E7300S
QuantSeq 3′ end Kit for Ion Torrent	Lexogen	012.24A
QuantSeq 3′ mRNA-Seq Library Prep Kit FWD	Lexogen	015.96
µMACS Streptavidin Kit	Miltenyi Biotech	130-074-101
NextSeq 500/550 High Output v2.5 kit	Illumina	20024907
4-thiouridine	Biosynth	NT06186
EZ-link HPDP-Biotin	Thermo Fisher	21341
DNase-I (RNase-free)	NEB	M0303S
E. coli Poly(A) Polymerase Kit	NEB	M0276S
SUPERase-In RNase Inhibitor	Thermo Fisher	AM2694

ThermoPol® Reaction Buffer Pack	NEB	B9004S
miRNeasy Micro Kit	Qiagen	217084
5-PRIME Phase Lock Gel Heavy	VWR	733-2478
GlycoBlue co-precipitant	Thermo Fisher	AM9515
Monarch Spin RNA Cleanup Kit	NEB	T2040L
BspQI	NEB	R0712
4-thio-UTP	Jena Bioscience	NU-1156L
HighYield T7 mRNA Synthesis Kit	Jena Bioscience	RNT-101
MEGAscript T7 Transcription kit	Thermo Fisher	AM1334
BioAnalyzer DNA High Sensitivity Kit	Agilent	5067-4626/27
BioAnalyzer RNA 6000 Pico kit	Agilent	5067-1513/14/35
TapeStation High Sensitivity RNA ScreenTape kit	Agilent	5067-5579/80/81
TapeStation RNA ScreenTape kit	Agilent	5067-5576/77/78
Qubit RNA High Sensitivity Assay Kit	Thermo Fisher	Q32852
Qubit RNA Broad Range Assay Kit	Thermo Fisher	Q10210
SYBR Gold Nucleic Acid Gel Stain	Thermo Fisher	S11494
RNA 5' Pyrophosphohydrolase (RppH)	NEB	M0356S
cOmplete, Mini, EDTA-free Protease Inhibitor Cocktail	Roche	11836170001
Pefabloc SC (AEBSF)	Roche	11585916001

Nextera XT DNA library prep kit	Illumina	FC-131-1096
MinElute PCR purification kit	Qiagen	28004
AxyPrep Mag PCR clean up kit	Appleton Woods	AX401
Anti-H3K4me3	Merck Millipore	05-745R
Anti-H3K27ac	Merck Millipore	07-360
Anti-CTCF	Cell Signalling Technology	3418S
Anti-H2AV Drosophila Ab	Active Motif	39715
PE Mouse IgG2b, Isotype Ctrl Antibody	Biolegend	400313

Software / Packages

Software / Package / Tool Name	Citation / Source
Trim-Galore	Babraham Bioinformatics
Trimmomatic	(9)
BBTools	https://sourceforge.net/projects/bbmap/
Fastqc	Babraham Bioinformatics
HISAT2	(28)
Bowtie2	(12)

STAR	(29)
Ion Torrent Server TMAP aligner	https://github.com/iontorrent/TMAP
Samtools	(6)
Bedtools	(8)
MACS2	(23)
UCSC utility tools	UCSC
featureCounts	(7)
Paraclu	(13)
TSScall	(14)
ggplot2	Hadley Wickham. ggplot2: Elegant Graphics for Data Analysis, 2016
Ggpubr	https://rpkgs.datanovia.com/ggpubr/
DESeq2	(25)
deepTools	(30)
PCAtools	https://github.com/kevinblighe/PCAtools
SPLICE-Q	(18)
Integrative Genome Viewer (IGV)	(31)
R	R Core Team. R: A Language and Environment for Statistical Computing, 2022.

Python	Guido Van Rossum and Fred L. Drake. Python 3 Reference Manual, 2009.
MATLAB	The MathWorks Inc.

1. Erickson, B., Sheridan, R.M., Cortazar, M. and Bentley, D.L. (2018) Dynamic turnover of paused Pol II complexes at human promoters. *Genes & development*, **32**, 1215–1225.
2. Tome, J.M., Tippens, N.D. and Lis, J.T. (2018) Single-molecule nascent RNA sequencing identifies regulatory domain architecture at promoters and enhancers. *Nat Genet*, **50**, 1533–1541.
3. Weltner, J., Balboa, D., Katayama, S., Bespalov, M., Krjutskov, K., Jouhilahti, E.M., Trokovic, R., Kere, J. and Otonkoski, T. (2018) Human pluripotent reprogramming with CRISPR activators. *Nat Commun*, **9**, 2643.
4. Schwalb, B., Michel, M., Zacher, B., Frühauf, K., Demel, C., Tresch, A., Gagneur, J. and Cramer, P. (2016) TT-seq maps the human transient transcriptome. *Science*, **352**, 1225–1228.
5. Kim, D., Paggi, J.M., Park, C., Bennett, C. and Salzberg, S.L. (2019) Graph-based genome alignment and genotyping with HISAT2 and HISAT-genotype. *Nature biotechnology*, **37**, 907–915.
6. Li, H., Handsaker, B., Wysoker, A., Fennell, T., Ruan, J., Homer, N., Marth, G., Abecasis, G., Durbin, R. and Genome Project Data Processing, S. (2009) The Sequence Alignment/Map format and SAMtools. *Bioinformatics (Oxford, England)*, **25**, 2078–2079.
7. Liao, Y., Smyth, G.K. and Shi, W. (2014) featureCounts: an efficient general purpose program for assigning sequence reads to genomic features. *Bioinformatics (Oxford, England)*, **30**, 923–930.
8. Quinlan, A.R. and Hall, I.M. (2010) BEDTools: a flexible suite of utilities for comparing genomic features. *Bioinformatics (Oxford, England)*, **26**, 841–842.
9. Bolger, A.M., Lohse, M. and Usadel, B. (2014) Trimmomatic: a flexible trimmer for Illumina sequence data. *Bioinformatics (Oxford, England)*, **30**, 2114–2120.
10. Roy, K., Gabunilas, J., Gillespie, A., Ngo, D. and Chanfreau, G.F. (2016) Common genomic elements promote transcriptional and DNA replication roadblocks. *Genome Res*, **26**, 1363–1375.
11. Amemiya, H.M., Kundaje, A. and Boyle, A.P. (2019) The ENCODE Blacklist: Identification of Problematic Regions of the Genome. *Sci Rep*, **9**, 9354.
12. Langmead, B. and Salzberg, S.L. (2012) Fast gapped-read alignment with Bowtie 2. *Nat Methods*, **9**, 357–359.
13. Frith, M.C., Valen, E., Krogh, A., Hayashizaki, Y., Carninci, P. and Sandelin, A. (2008) A code for transcription initiation in mammalian genomes. *Genome Res*, **18**, 1–12.
14. Lavender, C.A., Cannady, K.R., Hoffman, J.A., Trotter, K.W., Gilchrist, D.A., Bennett, B.D., Burkholder, A.B., Burd, C.J., Fargo, D.C. and Archer, T.K. (2016) Downstream Antisense Transcription Predicts Genomic Features That Define the Specific Chromatin Environment at Mammalian Promoters. *PLoS Genet*, **12**, e1006224.
15. Villamil, G., Wachutka, L., Cramer, P., Gagneur, J. and Schwalb, B. (2019) Transient transcriptome sequencing: computational pipeline to quantify genome-wide RNA kinetic parameters and transcriptional enhancer activity. *bioRxiv*, 659912.

16. Banks, C.A., Lee, Z.T., Boanca, G., Lakshminarasimhan, M., Groppe, B.D., Wen, Z., Hattem, G.L., Seidel, C.W., Florens, L. and Washburn, M.P. (2014) Controlling for gene expression changes in transcription factor protein networks. *Mol Cell Proteomics*, **13**, 1510–1522.
17. Day, D.S., Zhang, B., Stevens, S.M., Ferrari, F., Larschan, E.N., Park, P.J. and Pu, W.T. (2016) Comprehensive analysis of promoter-proximal RNA polymerase II pausing across mammalian cell types. *Genome Biology*, **17**, 120.
18. de Melo Costa, V.R., Pfeuffer, J., Louloupi, A., Ørom, U.A.V. and Piro, R.M. (2021) SPLICE-q: a Python tool for genome-wide quantification of splicing efficiency. *BMC Bioinformatics*, **22**, 368.
19. Fischl, H., McManus, D., Oldenkamp, R., Schermelleh, L., Mellor, J., Jagannath, A. and Furger, A. (2020) Cold-induced chromatin compaction and nuclear retention of clock mRNAs resets the circadian rhythm. *The EMBO journal*, **39**, e105604.
20. Wang, R., Nambiar, R., Zheng, D. and Tian, B. (2018) PolyA_DB 3 catalogs cleavage and polyadenylation sites identified by deep sequencing in multiple genomes. *Nucleic Acids Res*, **46**, D315–D319.
21. O'Leary, N.A., Wright, M.W., Brister, J.R., Ciufo, S., Haddad, D., McVeigh, R., Rajput, B., Robbertse, B., Smith-White, B., Ako-Adjei, D. *et al.* (2016) Reference sequence (RefSeq) database at NCBI: current status, taxonomic expansion, and functional annotation. *Nucleic Acids Res*, **44**, D733–745.
22. Buenrostro, J.D., Wu, B., Chang, H.Y. and Greenleaf, W.J. (2015) ATAC-seq: A Method for Assaying Chromatin Accessibility Genome-Wide. *Current protocols in molecular biology / edited by Frederick M. Ausubel ... [et al*, **109**, 21 29 21–21 29 29.
23. Zhang, Y., Liu, T., Meyer, C.A., Eeckhoute, J., Johnson, D.S., Bernstein, B.E., Nusbaum, C., Myers, R.M., Brown, M., Li, W. *et al.* (2008) Model-based analysis of ChIP-Seq (MACS). *Genome Biol*, **9**, R137.
24. Schmidl, C., Rendeiro, A.F., Sheffield, N.C. and Bock, C. (2015) ChIPmentation: fast, robust, low-input ChIP-seq for histones and transcription factors. *Nat Methods*, **12**, 963–965.
25. Love, M.I., Huber, W. and Anders, S. (2014) Moderated estimation of fold change and dispersion for RNA-seq data with DESeq2. *Genome Biol*, **15**, 550.
26. Wang, K., Wang, H., Li, C., Yin, Z., Xiao, R., Li, Q., Xiang, Y., Wang, W., Huang, J., Chen, L. *et al.* (2021) Genomic profiling of native R loops with a DNA-RNA hybrid recognition sensor. *Science advances*, **7**.
27. Blears, D., Lou, J., Fong, N., Mitter, R., Sheridan, R.M., He, D., Dirac-Svejstrup, A.B., Bentley, D. and Svejstrup, J.Q. (2024) Redundant pathways for removal of defective RNA polymerase II complexes at a promoter-proximal pause checkpoint. *Molecular Cell*, **84**, 4790–4807.e4711.
28. Kim, D., Paggi, J.M., Park, C., Bennett, C. and Salzberg, S.L. (2019) Graph-based genome alignment and genotyping with HISAT2 and HISAT-genotype. *Nature biotechnology*, **37**, 907–915.
29. Dobin, A., Davis, C.A., Schlesinger, F., Drenkow, J., Zaleski, C., Jha, S., Batut, P., Chaisson, M. and Gingeras, T.R. (2013) STAR: ultrafast universal RNA-seq aligner. *Bioinformatics (Oxford, England)*, **29**, 15–21.
30. Ramírez, F., Dündar, F., Diehl, S., Grüning, B.A. and Manke, T. (2014) deepTools: a flexible platform for exploring deep-sequencing data. *Nucleic Acids Research*, **42**, W187–W191.
31. Robinson, J.T., Thorvaldsdottir, H., Winckler, W., Guttman, M., Lander, E.S., Getz, G. and Mesirov, J.P. (2011) Integrative genomics viewer. *Nature biotechnology*, **29**, 24–26.
